# Towards carbon neutrality: Sustainable recycling and upcycling strategies and mechanisms for polyethylene terephthalate via biotic/abiotic pathways

**DOI:** 10.1016/j.eehl.2024.01.010

**Published:** 2024-02-27

**Authors:** Jiaqi Yang, Zhiling Li, Qiongying Xu, Wenzong Liu, Shuhong Gao, Peiwu Qin, Zhenglin Chen, Aijie Wang

**Affiliations:** a School of Civil & Environmental Engineering, Harbin Institute of Technology (Shenzhen), Shenzhen 518055, China; b State Key Laboratory of Urban Water Resources and Environment, School of Environment, Harbin Institute of Technology, Harbin 150090, China; c Institute of Biopharmaceutical and Health Engineering, Shenzhen International Graduate School, Tsinghua University, Shenzhen 518055, China; d Tsinghua-Berkeley Shenzhen Institute, Tsinghua Shenzhen International Graduate School, Tsinghua University, Shenzhen 518055, China

**Keywords:** PET depolymerization, Monomers, Recycling/upcycling, Biocatalysis, Photo/electrocatalysis

## Abstract

Polyethylene terephthalate (PET), one of the most ubiquitous engineering plastics, presents both environmental challenges and opportunities for carbon neutrality and a circular economy. This review comprehensively addressed the latest developments in biotic and abiotic approaches for PET recycling/upcycling. Biotically, microbial depolymerization of PET, along with the biosynthesis of reclaimed monomers [terephthalic acid (TPA), ethylene glycol (EG)] to value-added products, presents an alternative for managing PET waste and enables CO_2_ reduction. Abiotically, thermal treatments (i.e., hydrolysis, glycolysis, methanolysis, etc.) and photo/electrocatalysis, enabled by catalysis advances, can depolymerize or convert PET/PET monomers in a more flexible, simple, fast, and controllable manner. Tandem abiotic/biotic catalysis offers great potential for PET upcycling to generate commodity chemicals and alternative materials, ideally at lower energy inputs, greenhouse gas emissions, and costs, compared to virgin polymer fabrication. Remarkably, over 25 types of upgraded PET products (e.g., adipic acid, muconic acid, catechol, vanillin, and glycolic acid, etc.) have been identified, underscoring the potential of PET upcycling in diverse applications. Efforts can be made to develop chemo-catalytic depolymerization of PET, improve microbial depolymerization of PET (e.g., hydrolysis efficiency, enzymatic activity, thermal and pH level stability, etc.), as well as identify new microorganisms or hydrolases capable of degrading PET through computational and machine learning algorithms. Consequently, this review provides a roadmap for advancing PET recycling and upcycling technologies, which hold the potential to shape the future of PET waste management and contribute to the preservation of our ecosystems.

## 1 Introduction

The invention of plastics in the 20th century marked a significant technological milestone, specializing in versatile, hygienic, lightweight, flexible, and highly durable properties for various applications. Around 450 million tons of plastics have been substantially fabricated annually since the 1950s [1], leading to about 5 billion tons of plastics accumulating in environments like a “plastic time bomb” [2]. Plastics are typically long-lived materials characterized by covalent bonds (C–H, C–C, C–O) that are difficult to depolymerize naturally [3]. Plastic threats are a global concern due to their effects on the stability of the ecosystem, human health, and social activities. Increasing plastic proliferation threatens global climate change, with almost 1,800 megatons of CO_2_-equivalents in greenhouse gas (GHG) discharged in 2015, and an estimated 6,500 megatons of CO_2_-equivalents to be released by 2050 [4,5]. Exposure to microplastics (1 μm–5 mm) or nano-plastics (<1 μm) leads to adverse effects on living beings and environments [6,7]. Traditional treatments of plastics, including landfill, incineration, and mechanical recycling, are considered as downcycling pathways since they result in the wastes of sources (carbon, energy), environmental issues (i.e., leachates, toxicity, incomplete combustion), or diminished plastic qualities (Fig. S1). These traditional approaches cannot enable a fully circular plastics economy. Excessive plastic waste has exceeded the capability of countries or regions to deal with the associated risks. To address this challenge, upcycling (tertiary recycling) emerges as an ideal solution for plastic life cycle [8], which can depolymerize plastics into intermediates/products to synthesize plastics with properties akin to virgin materials (closed-loop recycling) or to produce value-added materials (open-loop upcycling) (Fig. S1).

Polyethylene terephthalate (PET), one of the most dominant plastic types, is the most used polyester and exemplifies hydrolyzable C–O polymers in backbone structure. PET constitutes 9%–10% of the plastics produced worldwide, with a global production of 70 million tons annually, used in textiles, packaging, and single-use beverage bottles [9]. The Market size of PET is projected to grow from $26.99 billion in 2024 to $36.61 billion by 2029, with a Compound Annual Growth Rate of 6.29% during the forecast period [10]. It exhibits 30%–40% crystallinity, a melting point (T_m_) of 255–265 °C, and glass transition temperatures (T_g_) of 60–80 °C [11]. Currently, only <20% of PET can be recycled by mechanical methods, leading to the deterioration of the material properties and value-decreased products for downcycling [12]. Through investigation, biotic depolymerization, using microbes and enzymes, provides a green and sustainable recycling pathway for PET with less energy consumption and lower CO_2_ emissions [8]. Additionally, chemical approaches can depolymerize PET into monomers and enable the potential generation of value-added products by pyrolysis and photo/electrocatalysis far beyond the scope of recycling. However, previous reviews on PET reuse focused on biological or chemical treatments and lacked systemic statements on PET transformation mechanisms based on categorizations of products or cycling patterns. This review aims to summarize the overall biological/chemical treatments for PET conversion and discuss the transformation pathways and mechanisms for different PET monomers. The context proposes to (1) highlight the advancements and challenges in developed biotic (biological) and abiotic (chemical) technologies aiming to achieve PET recycling/upcycling and economic circulation of resources; (2) explore the key conversion pathways and mechanisms in relevant biotic metabolism and abiotic transformation of PET polymers/intermediates; (3) discuss the challenges and opportunities of PET in current developments, as well as the prospects for sustainable plastic recycling/upcycling. The progressive developments in PET recycling/upcycling would significantly contribute to carbon neutrality in the circular economy, serving as guidelines or references.

## 2 Biotic pathways for PET recycling/upcycling

### 2.1 Biotic recycling of PET in closed-loop approaches

Biotic depolymerization of PET mainly benefits from the occurrence of PET-hydrolyzing enzymes, including PET surface-modifying enzymes and PET hydrolases (PETase) [13,14]. Surface-modifying enzymes, such as lipases, esterases, carboxylesterases, and papain, merely cause surface modification of PET materials with a depolymerization rate <1 wt% to 2 wt% [15]. PETase represents the enzymes that depolymerize PET at higher levels (>10 wt%), hydrolyzing ester bonds in the main body PET [16]. PETase firstly catalyzes PET to bis(2-hydroxyethyl) terephthalate (BHET) and mono(hydroxyethyl) terephthalate (MHET). Then, BHET hydrolases (BHETase) and MHET hydrolases (MHETase) further degrade BHET and MHET into terephthalic acid (TPA) and ethylene glycol (EG) [17]. Through investigation, the higher efficient PETases (i.e., cutinases and *Is*PETase) are specifically reviewed to promote resource circularity.

#### 2.1.1 Cutinases for PET hydrolyzing

At present, four main types of cutinases (from actinomycetes and fungus) have been categorized as PETases that can significantly hydrolyze PET, which has shown a superior thermostability (T_m_ of 53.9–98.1 °C) (Table 1): (1) Cutinases from *Thermobifida fusca* (TfH and TfCut2) resulted in 42%–50% weight loss of PET at 55–70 °C with >50 h incubation [[18], [19], [20]]; (2) A cutinase from *Saccharomonospora viridis* (Variant of Cut190) performed higher thermostability that can depolymerize PET films at 70 °C [21]; (3) A cutinase from *Humicola insolens* (HiC) showed significant 97% ± 3% of weight losses of PET in 96 h [22]; and (4) Wild-type leaf-branch compost metagenome (LCC) and the variants (ICC, WCC, ICCG, ICCM, WCCG, and WCCM), some showed the ability to effectively exhibit >90% weight loss of PET within 10 h at 72 °C [23]. Additionally, two metagenome-derived thermophilic polyester hydrolases, PHL7 and BhrPETase, have gained attention for their superior performance in PET bio-conversion [23,24]. PHL7, for example, can enzymatically hydrolyze PET at 65–70 °C, achieving >90% weight loss of PET films within 16 h [25]. BhrPETase, with 94% sequence identity to LCC, is the most thermostable PETase discovered, with a T_m_ of 101 °C [26].

**Table 1:** The efficient, optimized, and representative PETases.

Enzyme	Source	Optimized T (°C)	T_m_ (°C)	Degradation degree (measurements)	Reaction duration	PET used	Ref.
**Cutinase**							
TfH	*Thermobifida fusca*	55	–	∼50% (weight loss)	3 weeks	Melt pressed beverage bottle	[18]
Variant of TfCut2		65–70	80.7	42%–45% (weight loss)	50–96 h	Amorphous film (PET-Goodfellow)	[19,20]
Variant of Cut190	*Saccharomonospora viridis* AHK190	70	72.4	33.6% ± 3.0% (absorption)	1 week	PET-Goodfellow	[21]
HiC	*Humicola insolens*	70	–	97% ± 3% (NaOH)	96 h	PET-Goodfellow	[22]
LCC	Metagenome from plant compost	50–70	86.2	≤25% (weight loss)	18–48 h	PET package	[113]
LCC		72	84.7	53% (weight loss)	20 h	PET-Goodfellow	[23]
Variants of LCC		65	90.9–98.1	>90% (weight loss)	10–20 h	PET-Goodfellow	[23]
ICCG and WCCG variants of LCC		30–70	53.9–92.57	–	96–168 h	PET-Goodfellow; crystalline PET powder	[114]
***Is*PETase**							
*Is*PETase	*I. sakaiensis* 201-F6	30	–	Complete degradation	6 weeks	PET film (1.9% crystallinity)	[27]
Variant *Is*PETase	*I. sakaiensis* 201-F6	30	56.5	10.1% (crystallinity reduction)	96 h	PET film (15% crystallinity)	[115]
ThermoPETase	*I. sakaiensis* 201-F6	40	57.6	Complete degradation	24–72 h	PET film (41.79% crystallinity)	[30]
HotPETase	*I. sakaiensis* 201-F6	65–70	82.5	∼82%	24 h	Amorphous PET film	[29]
HotPETase	*I. sakaiensis* 201-F6	65–70	82.5	∼13%	24 h	Crystalline PET powder	[29]
BurPL-DM hydrolyses	*Burkholderiales bacterium*	35	58.6	Lower than *Is*PETase	18 h	PET-Goodfellow	[116]
DuraPETase	*I. sakaiensis* 201-F6	37	77	Complete degradation	10 d	PET microplastics (30% crystallinity)	[31]
FAST-PETase	*I. sakaiensis* 201-F6	50	67.4	96.8% (TPA yield) 22%–28% (weight loss)	96 h	PET films-Goodfellow	[24,117]
**Others**							
PHL1–PHL7	Plant compost metagenome	65–70	67.6–79.1	∼90% (weight loss)	16 h	PET-Goodfellow;	[25,118]
BhrPETase	Hotspring water metagenome	70	101	16 ± 0.8 μmol/(min·mg) (BHET yield)	20 h	Amorphous PET powder (11.2% crystallinity)	[26]

#### 2.1.2 *Is*PETases for PET hydrolyzing

*Ideonella sakaiensis* (*I. sakaiensis*) 201-F6 strain, initially reported by Yoshida et al. [27], can specifically depolymerize PET film (1.9% of crystallinity) at 30 °C within 6 weeks, revealing a naturally evolved PET-hydrolysing enzyme—*Is*PETase. However, wild-type *Is*PETase (*Is*PETaseWT) has limited applications due to the poor thermostability (T_m_ of 45 °C, lower than T_m_ of cutinases: 53.9–98.1 °C), reduced durability, and low enzymatic efficiency [28]. It exhibits reduced enzyme activity at temperatures > 40 °C, loses activity within 24 h at 37 °C, and takes weeks or months for complete PET degradation [29]. Since then, more studies on improving *I*sPETase performances by protein engineering have flooded in. The variants of *Is*PETase^WT^, such as ThermoPETase, DuraPETase, and FAST-PETase (Table 1), are performed with enhanced thermostability, durability, and hydrolyzing efficiency through rational design or computational assistance [30]. For example, ThermoPETase (*Is*PETase^S121E/D186H/R280A^), created via structure-based rational design, was reported to increase T_m_ by 8.81 °C [30]. The computer-designed DuraPETase (*Is*PETase^S214H/I168R/W159HS188Q/R280A/A180/G165A/Q119Y /L117F/T140D^) was reported to increase T_m_ by 31 °C, enhance PET degradation rate by over 300-fold at 37 °C, and prolong enzymatic activity for 10 days [31]. A quadruple variant *Is*PETase^S121E/D186H/S242T/N246D^ was reported to significantly extend enzymatic durability to 20 days at 37 °C [32]. Moreover, the most thermostable DuraPETase by Brott et al. [28] was reported to increase T_m_ to 81.1 °C by adding a disulfide bond. FAST-PETase, mutated from ThermoPETase via a machine-learning algorithm, can inclusively degrade >50 types of PET commodities (crystallinity of 2%–7%) in 1–2 weeks at 50 °C [24]. Besides engineering renovations, *Is*PETase can be subjected to directed evolution. HotPETase was screened from >13,000 mutants due to its outstanding enzymatic activities and T_m_ (82.5 °C). When increasing the temperature to 65–70 °C, the thermostability of HotPETase can reach a level as high as LCC and TfCut2 [29]. Although cutinase-based PETases generally exhibit higher thermostability than *Is*PETase, their non-optimized substrate binding sites significantly decrease PET degradation activity at mild temperatures, resulting in lower activity than wild/mutant *Is*PETases. Therefore, engineered *Is*PETase mutants with higher thermostability, durability, and hydrolyzing efficiency are highly desired for large-scale PET treatment.

These remarkable achievements suggest a promising future for industrial biocatalysis of PET. Two representatives of PETases have been identified with superior thermostability, durability, and hydrolyzing behaviors. However, PET hydrolyzing by PETase remains in closed-loop recycling; hence, advanced biotic/abiotic conversion must be explored for open-loop upcycling. Given the vast microbial biodiversity on earth, more microbes and enzymes capable of PET depolymerization can be discovered in nature. Where applicable, large-scale production of enzymes at low cost is crucial for sustainable recycling in the future.

### 2.2 Biotic upcycling of PET/PET monomers in open-loop approaches

Regarding bio-upcycling, the most common PET hydrolysates (TPA and EG) can be bio-converted by TPA/EG-metabolizing microorganisms (i.e., *Pseudomonas*, *Escherichia*, *Bacillus*) to produce high-value-added chemicals. Other PET hydrolysates, such as MHET and BHET, are first converted into TPA and EG and then processed through biocatalysis to produce valuable products [33]. However, TPA and EG need separate pathways to enter the cell's primary metabolic networks for further metabolism. Depending on substrates, the bio-upcycling approaches of PET can be categorized into EG-based, TPA-based, and TPA-EG-based bio-pathways. These pathways facilitate the integration of PET hydrolysates into microbial metabolic networks and ultimately result in valuable compounds. For more detailed cases with conversion mechanisms and end-products, please refer to Table S1.

#### 2.2.1 EG-based bio-pathways for upcycling

**Fig. 1:**
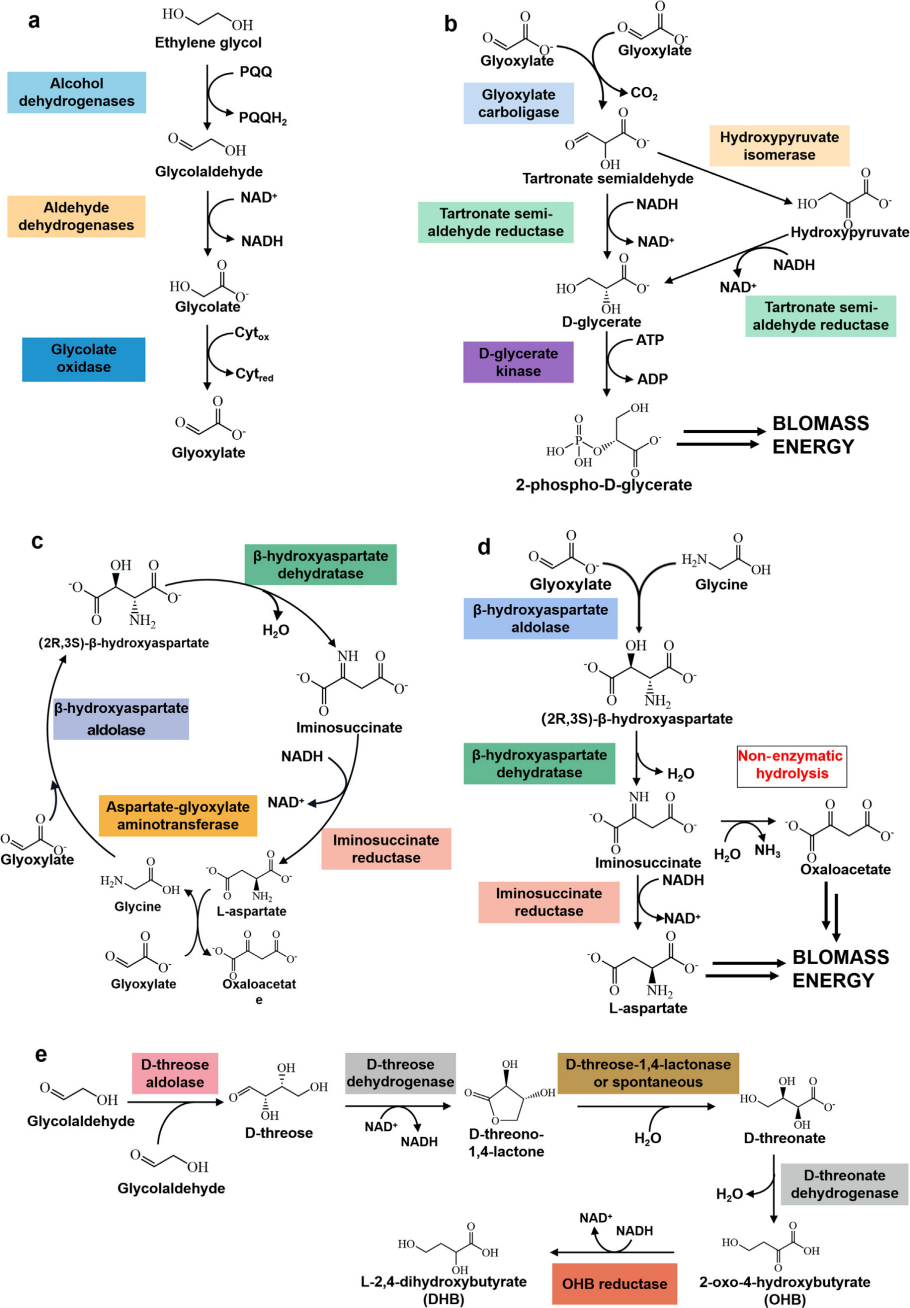
Microbial metabolism of EG for upcycling conversion. (a) Oxidative reactions from EG to glyoxylate. (b) The glycerate pathway, which converts two molecules of glyoxylate into 2-phosphoglycerate and CO_2_. (c) The β-hydroxyaspartate cycle using four enzymes to convert two molecules of glyoxylate into one molecule of oxaloacetate. (d) The β-hydroxyaspartate shunt converts glyoxylate and glycine into aspartate. (e) The synthetic DHB pathway using two molecules of glycolaldehyde derived from EG to synthesize DHB. (a)–(d) reproduced with permission from ref. [36]. Copyright 2023 Elsevier. (e) Reproduced with permission from ref. [39]. Copyright 2023 Springer Nature.

EG is an attractive two-carbon alcohol substrate that can be assimilated through natural tartronic semialdehyde pathways or non-natural acetyl-CoA-yielding routes (Fig. 1) [34,35]. *Pseudomonas putida* possesses the genetic inventory for several pathways that enable EG metabolism. EG can be metabolized stepwise into glycolaldehyde, glycolate, and glyoxylate (Fig. 1a) [36]. These conversions are catalyzed by alcohol/aldehyde dehydrogenases and glycolate oxidase, employing different cofactors such as NAD^+^, pyrroloquinoline quinone (PQQ), or cytochromes that funnel the electrons into the electron transport chain. Naturally, glyoxylate can further be assimilated in engineered strains (i.e., *P. putida* KT2440) via a glycerate pathway (Fig. 1b). However, this conversion only reduces equivalents without contributing to cell growth [37]. The EG assimilation to glycerate can be further improved via overexpression of glycolate oxidase genes and the glyoxylate carboligase (*Gcl*) operon encoding for glycerate pathway [34], or deletion of GclR (transcriptional regulator of *Gcl*) and overexpression of glycolate oxidase operon [33]. However, EG assimilation via the glycerate pathway is accompanied by the release of CO_2_, which lowers carbon efficiency and wastes, reducing equivalents. A novel β-hydroxyaspartate cycle was created to replace the glycerate pathway with a glyoxylate assimilation pathway, significantly improving the EG conversion [36,38]. On the one hand, the β-hydroxyaspartate cycle was reported to employ four enzymes (β-hydroxyaspartate aldolase, β-hydroxyaspartate dehydratase, iminosuccinate reductase, and aspartate-glyoxylate aminotransferase) to convert glyoxylate (two molecules) into oxaloacetate (Fig. 1c) [38]. On the other hand, the heterologous β-hydroxyaspartate cycle was reported to convert glyoxylate with glycine into aspartate, constituting a linear metabolic module terming β-hydroxyaspartate shunt (Fig. 1d). Moreover, glycolaldehyde can serve as an intermediate to synthetically convert EG into 2,4-dihydroxybutyric acid (DHB) which is an important precursor for methionine analogue 2-hydroxy-4-(methylthio)butyrate or 1,3-propanediol [39,40]. For DHB generation, five enzymes (d-threose aldolase, d-threose dehydrogenase, D-threono-1,4-lactonase, d-threonate dehydratase, and 2-oxo-4-hydroxybutyrate reductase) participate in the carbon-conserving biosynthesis of DHB from EG (Fig. 1e) [39]. Through investigation, the glyoxylate assimilation via the glycerate pathway or β-hydroxyaspartate cycles and the extended glycolaldehyde assimilation via the DHB pathway are the main metabolic routes of EG. These studies indicate great potential for converting the sustainable carbon source EG into a value-added compound of considerable industrial interest.

#### 2.2.2 TPA-based bio-pathways for upcycling

**Fig. 2:**
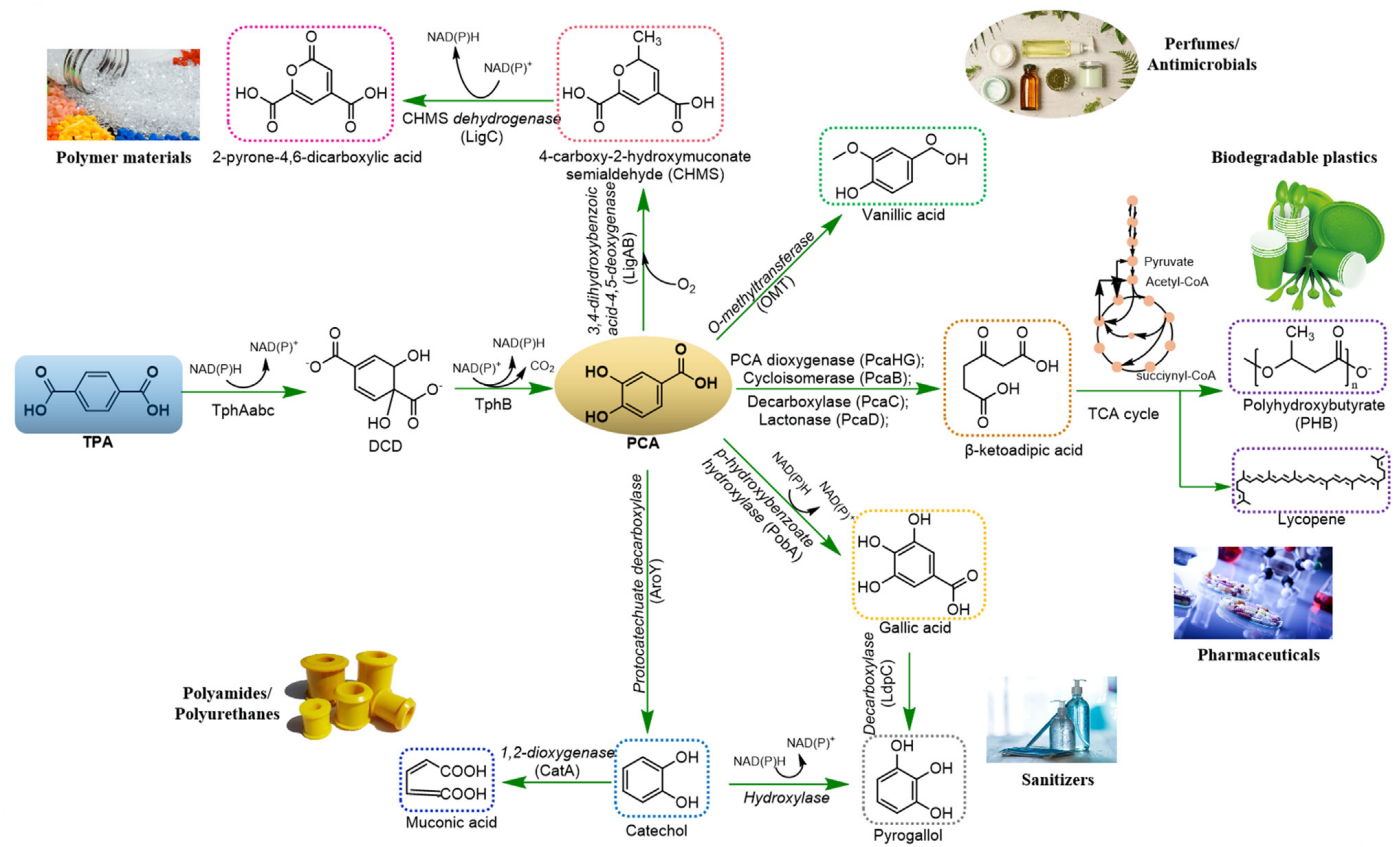
Microbial metabolism of TPA for upcycling conversion.

In TPA-based bio-pathways, monomeric TPA is metabolized to TPA-1,2-cis-dihydrodiol (DCD) via TPA-1,2-dioxygenases (TphAabc) and subsequently converted into protocatechuic acid (PCA) via TPA-1,2-cis-dihydrodiol dehydrogenase (TphB). These pathways, predominantly involving whole-cell bioconversion, have been identified in various genera, such as *Comamonas*, *Ideonella*, *Pseudomonas*, and *Rhodococcus jostii* (Fig. 2) [41]. Native TPA catabolism can lead to the production of a range of valuable aromatic or aromatic-derived chemicals [e.g. PCA, gallic acid (GA), pyrogallol, catechol, muconic acid (MA), vanillic acid (VA), 2-pyrone-4,6-dicarboxylic acid (PDC), and adipic acid] via certain wild-type or mutated hydroxylase, decorboxylase, dioxygenase, methyltransferase, etc. (Fig. 2) [[42], [43], [44], [45]]. The microbial synthesis of valuable products directly from PET remains challenging. However, Wallace et al. [45] first reported a one-pot bioproduction of adipic acid from waste PET in engineered *E.coli* DD-2. Moreover, TPA can be metabolized to acetyl-CoA and succinyl-CoA, which can fuel the tricarboxylic acid (TCA) cycle via the β-ketoadipate pathway and synthesize other chemicals, such as the acetyl-CoA derivatives [i.e., polyhydroxyalkanoates (PHA) and lycopene] [33,46]. For instance, an engineered *Pseudomonas stutzeri* TPA3P with a recombinant plasmid containing polyhydroxybutyrate synthesis genes (*phbCAB*) operons from *Ralstonia eutropha* was demonstrated to transfer TPA to polyhydroxybutyrate (PHB), a type of PHA [47].

#### 2.2.3 TPA-EG-based bio-pathways for upcycling

**Fig. 3:**
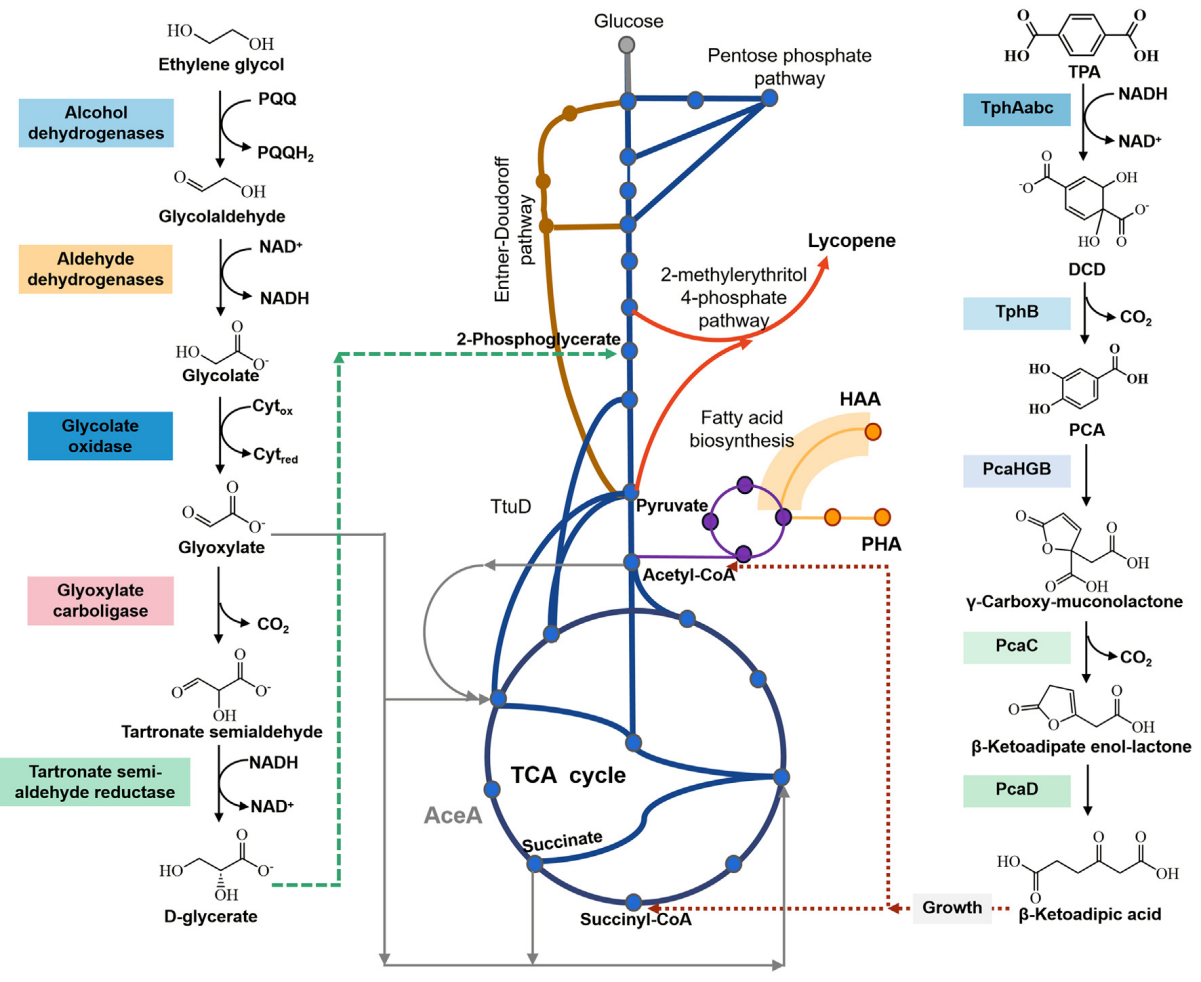
Metabolic pathways for the bioconversion of EG and TPA. Green lines represent the enzymatic steps of EG conversion to glycerate. Gray lines represent pathways in which EG serves only as a source for redox equivalents and the generation of CO_2_. Blue lines show the conversion of TPA to succinate and acetyl-CoA via TCA cycle. Reproduced with permission from ref. [51]. Copyright 2021 Elsevier.

In TPA-EG-based pathways, both TPA and EG are involved in microbial metabolisms. EG can serve as the carbon source for cell growth and can be naturally assimilated through the glyoxylate pathway, funneling into the TCA cycle via glyoxylate shunt [48,49]. TPA can be converted into acetyl-CoA and succinate to fuel the TCA cycle via the β-ketoadipate pathway [50]. Notably, when an alternative carbon source exists, EG only serves as an energy donor [48]. Tiso et al. [51] first demonstrated the effective conversion of TPA and EG into PHA using engineered *Pseudomonas umsongensis* GO16 KS3 (Fig. 3). It revealed a significant increase in EG depletion rate by using a synthetic mixture of TPA and EG compared to using EG alone. Moreover, this engineered *P. umsongensis* GO16 KS3 has been further modified to support the extracellular conversion of TPA and EG into hydroxy alkanoyl oxy-alkanoate (HAA), serving as the monomers for chemo-catalytic polymerization of bio-based poly(amide urethane) (Fig. 3) [51,52]. Another engineered *R. jostii* strain was reported to use TPA and EG as carbon sources to synthesize lycopene, fueling the TCA cycle via the β-ketoadipate pathway [50]. As a result, the direct conversion of EG might be caused by its high degree of reduction, providing enough redox cofactors for the expensive fatty acid *de novo* synthesis. Direct conversion of TPA cannot provide sufficient redox equivalents, suggesting the pentose phosphate pathway and TCA cycle as preferable routes for chemical synthesis [51].

## 3 Abiotic pathways for PET recycling/upcycling

### 3.1 Abiotic recycling of PET in closed-loop approaches

The manufacture of PET is derived from EG either with TPA by esterification or with dimethyl terephthalate (DMT) by transesterification and polycondensation. Numerous studies demonstrated the oriented depolymerization of PET into monomers and oligomers via abiotic approaches, primarily driven by the cleavage or substitution of ester bonds by molecules like water, EG, methanol, etc. In abiotic pathways, thermochemical treatments (i.e., hydrolysis, glycolysis, aminolysis/ammonolysis, and methanolysis) provide effective depolymerization of PET in closed-loop recycling (Fig. S2).

#### 3.1.1 PET hydrolysis

PET hydrolysis can be carried out in an acidic, alkaline, or neutral (water) medium at higher temperature and/or pressure, forming the monomers of TPA and EG [53]. Alkaline hydrolysis is mostly used for PET recycling, with an aqueous solution of NaOH or KOH (1 wt%–20 wt%) at the temperature of 40–260 °C, pressure of 101 kPa–3 MPa (Table S1) [[54], [55], [56]]. Alkaline hydrolysis is a first-order process concerning the OH^−^ concentration, and its activation energy in water is lower than that of neutral heterogeneous hydrolysis [57]. Meanwhile, phase-transfer catalysts (PTCs) can further accelerate the collision among the base and solid-state polymer molecules, increasing its yield and *ε* (energy economy factor) [58]. Ru/mesoporous carbon was used as PTC to completely degrade PET within 2 h under 240 °C, 2 MPa [56]. In alkaline hydrolysis and ultrasound treatment, tetrabutyl ammonium iodide as PTC reduced the depolymerization duration to 45 min at 90 °C [59]. Furthermore, certain organic solvents (i.e., EG and ethanol) improve alkaline PET hydrolyzing conditions. For instance, the addition of EG resulted in 95% degradation of PET within 35 min at 100 °C [60]. The addition of 60 vol% of ethanol achieved >92% degradation of PET within 20 min at 80 °C [61].

#### 3.1.2 Glycolysis and methanolysis

Glycolysis can depolymerize PET to BHET in EG environments via transesterification, which can achieve 70%–100% yielding of BHET within 10–180 min at atmospheric pressure and temperatures of 190–300 °C [58]. For a long time, catalysts have played an essential role in PET glycolysis, including metallic catalysts, organocatalysts, polyoxometalates, and ionic liquid-based catalysts. Generally, metallic catalysts for glycolysis are synthetically reviewed by Xin et al. [62], including organometallic, metal acetates [i.e., Pd(OAc)_2_, Zn(OAc)_2_, Co(OAc)_2_, Mn(OAc)_2_], metal-oxides (i.e., Zeolites, Mn_3_O_4_, Fe_3_O_4_), and metallic, ionic liquid catalysts (i.e., CuCl_2_, FeCl_3_, MnCl_2_, ZnCl_2_) which are specialized in optimizing reactivity and selectivity of reactions. Organocatalysis (i.e., triazabicyclodecene (3-amino-propyl)-tributyl-phosphonium glycine/alanine, choline-based organocatalysts) significantly promotes polymer depolymerization as well as polymer synthesis [62,63]. Using 1,3-dimethylimidazolium-2-carboxylate as an organocatalyst accelerated the depolymerization of PET in less than 1 h at 180 °C, thanks to the formation of nucleophilic N-heterocyclic carbene [64]. Additionally, solar-thermal catalysis, such as using polydopamine-modified carbon nanotubes as catalysts, promotes glycolysis behaviors, allowing PET depolymerization at 150 °C while similar reactions occurred at 180 °C by glycolysis alone [65].

Methanolysis of PET yields DMT and EG at 25–280 °C. Similarly, as a form of transesterification, methanolysis proceeds effectively with catalysts, such as metal salts, metal oxides, and organocatalysts. For instance, when using aluminum tiisopropoxide as the catalyst for PET methanolysis at 200 °C, the yields of DMT and EG achieved 64% and 63%, respectively [66]. A low-energy catalytic route for methanolysis using K_2_CO_3_ as a catalyst was demonstrated to obtain 93.1% yielding of DMT at 25 °C within 24 h [67]. However, since methanolysis generates additive by-products (i.e., glycols, alcohols, phthalate derivatives), it is considered a costly process requiring both depolymerization and product separation [68].

#### 3.1.3 Ammonolysis and aminolysis

Ammonolysis of PET, conducted with ammonia in an EG environment, yields terephthalamide under a pressure of ∼2 MPa, temperature of 70–180 °C for 1–7 h [69]. Aminolysis of PET uses amine aqueous (i.e., methylamine, ethylamine, ethanolamine) as a nucleophile to attack ester groups and cleave PET chains at 20–100 °C, yielding diamides of TPA [69]. Aminolysis alone results in incomplete depolymerization of PET and complex by-products [55]. When assisted by homogeneous catalysis or complex homogeneous catalysts, it significantly improves the stability and yields of aminolysis. Among all thermal treatments, aminolysis is the least studied due to the unavailable formation of amide products. Nevertheless, the aminolysis process introduces a nitrogen source (residual ammonium ions from processing and nitrogen in diamides), replacing what would be present in microbial media, potentially fitting for hybrid thermochemical-biological processing [70]. Another attractive pathway of aminolysis to produce value-added products is the synthesis of poly(ester amides), revealing it as a potential pathway for open-loop upcycling of PET [71].

#### 3.1.4 Thermal intensification approaches

To further achieve complete PET depolymerization by thermochemical approaches, a series of intensification methods have been introduced, such as supercritical intensification, microwave intensification, and cosolvent intensification [72]. Microwave-assisted saponification is a super-fast method that can completely depolymerize PET to Na_2_-TPA within 2 min in an alkaline environment [73]. Methanolysis under super criticality can significantly shorten the PET degradation duration to 1 h, achieving approximately 90% yield of DMT at 9–11 MPa, 260–270 °C [74]. Microwave-assisted methanolysis, using zinc acetate as a catalyst, shortened the reaction time to 30 min at a milder temperature of 160 °C, resulting in 100% degradation of PET and 80% yield of DMT [75]. A cosolvent (acetonitrile)-enhanced methanolysis of PET with deep eutectic solvents as catalysts achieved 100% conversion of PET at 120 °C within 2 h. Microwave-assisted glycolysis, using ZnO as a heterogeneous catalyst, achieved >95% yields of BHET in <10 min at 210 °C [76]. While intensification methods significantly achieve rapid depolymerization of PET in shorter periods, they usually demand higher temperatures and pressures, and more sophisticated reaction equipment. Overall, thermochemical approaches highly accelerate the depolymerization processes of PET, mainly based on the hemolysis occurring at the polymer backbone by nucleophiles (methanol, water, EG, etc.). Through investigation, each approach from above can convert PET in a directional pathway, including hydrolysis (PET→TPA + EG), glycolysis (PET→BHET), aminolysis/ammonolysis (PET→ammoniated TPA + EG), and methanolysis (PET→DMT + EG). Additionally, optimizing inorganic or organic catalysts is essential for PET's depolymerization performances. Specifically, the use of organic phases such as toluene and xylene can increase the solubility of oligomeric compounds produced during thermal reaction, thereby enhancing the PET conversion. Through these investigations, it has been shown that PET can be directionally transformed into dimers or monomers (i.e., BHET, DMT, TPA, EG), contributing to PET resynthesis in closed-loop recycling. However, further transformations are significantly needed for open-loop upcycling.

### 3.2 Abiotic upcycling of PET/PET monomers in open-loop approaches

For upcycling, further conversion of PET/PET monomers via photocatalysis, electrocatalysis, or thermal treatments is expected to form valuable fuels or feedstocks or other value-added chemicals, such as gaseous (i.e., H_2_ and syngas), liquid (i.e., acids and fuels), and solid (carbonaceous materials) products (Table S1). Specifically, the advanced oxidation strategies (i.e., photocatalysis, electrocatalysis) have attracted extensive attention to the oxidative transformation of EG due to their environmentally friendly concepts and efficient redox activities.

#### 3.2.1 Thermochemical approaches to upcycling

In addition to closed-loop recycling, thermochemical approaches can also convert PET to value-added products for upcycling. Aminolysis and ammonolysis of PET produce diamides/amides of TPA, which can be used as plasticizers, adhesives, antibacterial drugs, textile dyes, etc. [55]. Diamides of TPA can be further synthesized to poly(ester-amide)s via melt polycondensation [71]. Furthermore, amides of TPA can serve as precursors to produce terephthalonitrile, *p*-xylylenediamine, and 1,4-bis-aminoethyl cyclohexane, which can be used as organic solvents and chemical synthesis intermediates (i.e., dyes, pesticide, emulsifier) [77]. Additionally, PET-to-DMT-to-*p*-xylene (fuel) by methanolysis over Cu/SiO_2_ catalyst has been achieved [78], carried out by tandem PET methanolysis and DMT hydrodeoxygenation at 210 °C. Another pathway of PET to dioctyl terephthalate (plasticizer) was achieved by alcoholysis using 2-ethyl-1-hexanol as solvent [79]. Through glycolysis, PET can be first glycolyzed with diol, then converted into unsaturated polyester or vinyl ester with renewable monomers (e.g., olefinic acid), and finally produce fiber-reinforced plastics [80]. Similarly, Karanastasis et al. [81] combined the pristine BHET or postconsumer PET with a hydrophobic dimer fatty acid to synthesize segmented thermoplastic copolyesters via solvent-free melt polycondensation. However, as the base monomers of PET, TPA is rarely converted by thermochemical methods, while EG conversions (e.g., acidification, substitution, oxidation) are mostly preferred for upcycling. For instance, considering the lower solubility of TPA in acetic acid, a new strategy was developed to crystallize out TPA in high purity and to synthesize ethylene glycol diacetate via PET acetolysis in acetic acid [82]. Cao et al. [83] creatively developed a co-upcycling strategy of PVC and PET via an anhydrous thermal-catalytic process, where the in situ PVC-released chloride could be preserved in ionic liquid-Bu_4_PCl solvent, then the generated chloride ions/HCl would attack the C_alkyl_–O bond in PET via nucleophilic substitution to produce 1,2-dichloroethane and TPA, under the combined action of Bu_4_PCl and ZnCl_2_ catalyst.

#### 3.2.2 Photo/electrocatalysis to upcycling

**Fig. 4:**
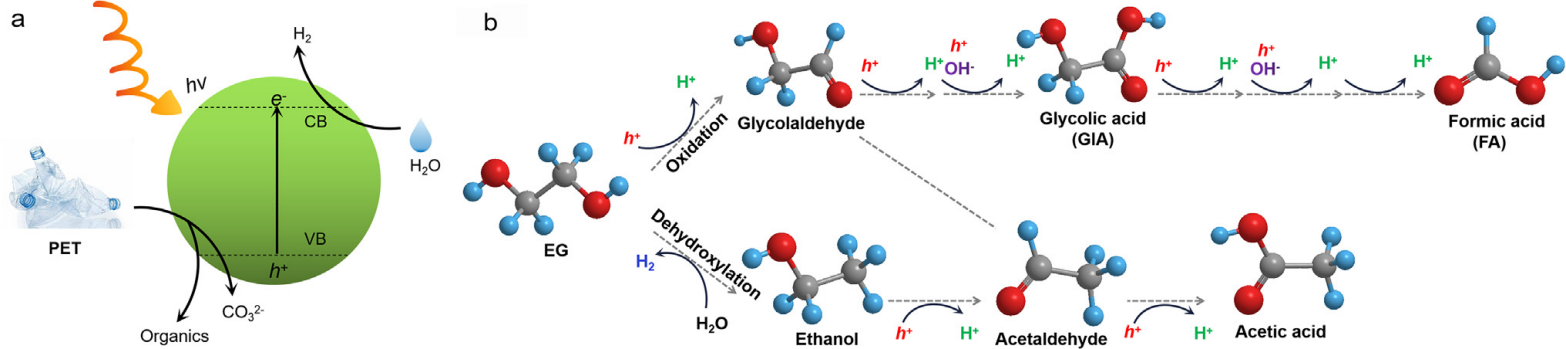
(a) Diagram of the PET photoreforming process via photocatalysts. Reproduced with permission from ref. [85]. Copyright 2018 Royal Society of Chemistry. (b) Photoforming pathways of EG [119], element color: O-Red; C-Gray; H-Cyan.

Photocatalysis is an environmental and economic technique for plastic reclamation. By sunlight absorption, the photocatalyst excites electron-hole pairs, promoting the reduction of H_2_O to H_2_ as photoinduced electrons move to the conduction band (CB). Simultaneously, the remaining photogenerated holes in the valence band (VB) oxidize the substrate-plastics (i.e., EG) to smaller organic molecules. In this process, plastics serve as the sacrificed electron donor, facilitating H_2_O reduction (Fig. 4a). Photoreforming is replacing the energetically demanding oxygen evolution reaction with oxidation of other substrates to enable the co-production of valuable chemicals and H_2_ fuel. Remarkably, the photoreforming of EG can produce H_2_ and organic products (e.g., glycolaldehyde, formate, glycolate, ethanol, acetate) (Fig. 4b) [84,85], while TPA is hardly converted chemically. Therefore, the oxidation products of PET originate exclusively from its aliphatic components [85]. The selection of suitable photocatalysts is crucial to the photocatalytic efficiency. The photocatalysts, such as Pt/TiO_2_, carbon nanotubes-carbon nitride hybrids (CN-CNTs-NM), carbon nitride/nickel phosphide (CN_x_/Ni_2_P), carbonized polymer dots-graphitic carbon nitrid (CPDs-CN), and CdS/CdO_x_ quantum dots have been illustrated to effectively accelerate PET hydrolysis and generate H_2_ with the yields of 0.074 ± 0.029 mmol/(g_catalyst_·h) [85], 0.09 mmol/(g_catalyst_·h) [86], 0.046 ± 0.006 mmol/(g_catalyst_·h) [84], 1.03 ± 0.13 mmol/(g_catalyst_·h) [57], 3.42 ± 0.87 mmol/(g_catalyst_·h) [85], respectively. To further enhance PET conversion efficiency, the material Zn_x_Cd_1−x_S (Z_x_C_1−x_S, 0 ≤ x ≤ 1), which exhibits an appropriate band gap, good optical properties, and high utilization of visible light, is considered a promising photocatalyst [87]. By photocatalysis via Zn_x_Cd_1−x_S, PET is successfully converted into formate, methanol, and acetate with H_2_ yields of 6.68 mmol/(g_catalyst_·h) under visible light [88]. Moreover, 2D materials have garnered enormous interest in optoelectronic integration due to their ultrathin body, strong light–matter interactions, and compatibility with the current silicon photonic technology. Typical 2D materials include transition metal carbides and nitrides (MXenes), MoS_2_, and graphene, etc [89]. The extended MXene/Zn_0.6_Cd_0.4_S and MoS_2_/Zn_0.5_Cd_0.5_S photocatalysts further enhanced the separation abilities of photocarriers, redox abilities via optimum band structures, and light absorption, resulting in significant increase in H_2_ evolution rates of 14.17 and 15.90 mmol/(g_catalyst_·h), respectively [88,90]. Photoforming of EG yields H_2_ while TPA cannot, suggesting the oxidation products of PET originate exclusively from EG, such as formate, glyoxal, glycolate, acetate, etc (Fig. 4b). However, photoreforming of wastes often generates a portfolio of oxygenates and CO_2_, which is considered as nonselective degradation pathway. For upcycling, the selective synthesis of value-added products remains challenging and needs continuous breakthroughs.

**Fig. 5:**
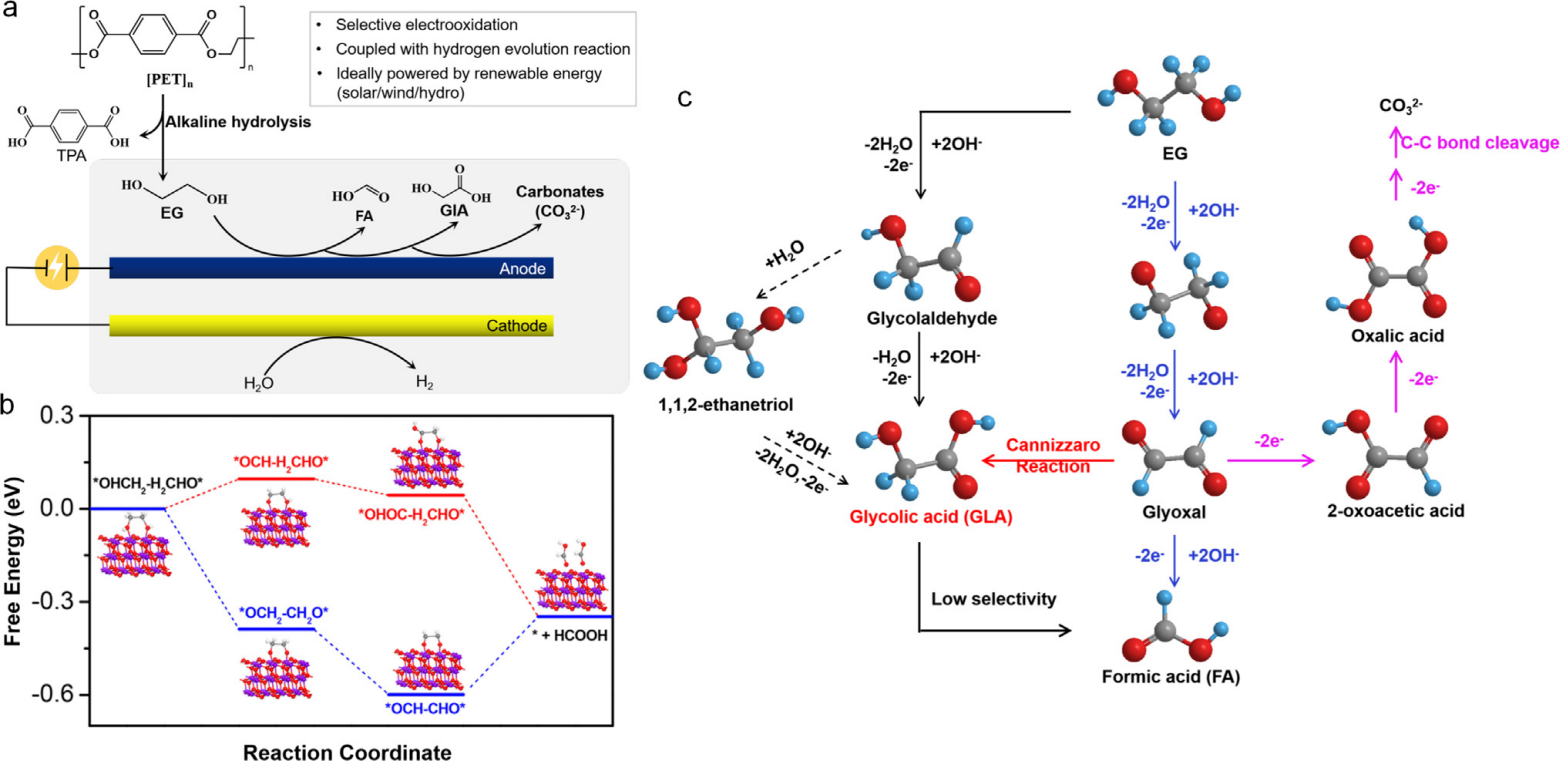
(a) Scheme of electrocatalytic conversion of PET hydrolysate (EG) to value-added chemicals and H_2_. (b) Reaction free energy diagram of EG-to-FA on anodic electrocatalysts by different pathways. Reproduced with permission from ref. [94]. Copyright 2022 American Chemical Society. (c) Proposed reaction pathways for the oxidation of EG to formic acid (Blue line), glycolic acid (Dashed black line), and carbonates (Purple line) in alkaline environments [96]. Element color: O-Red; C-Gray; H-Cyan.

Electrocatalysis, powered by renewable energy (solar, wind, and hydro), represents a sustainable and attractive strategy to stimulate redox reactions and generate valuable products by accelerating electron transfers [91,92]. The foremost challenge for the electrocatalysis of plastics is how to enhance the interaction between the electrode surfaces and the macromolecule of plastics to ensure efficient electron transfer. The ester bonds in PET are hydrolyzed in alkali media to yield TPA and EG, perfectly compatible with alkali water electrolysis, leveraging the electrooxidation of EG-to-valuable oxygenates [i.e., formate, formic acid (FA), glycolic acid (GLA), glycolate, acetate, etc.] coupled with H_2_ production (Fig. 5a). The implementation of electrocatalytic oxidation requires sufficient voltage, electrocatalyst, reaction substrate, and suitable electrolyte, while current researchers are more likely to focus on the selection and optimization of electrocatalysts. For instance, a CoNi_0.25_P electrocatalyst was developed to convert EG-to-formate at 500 mA/cm^2^ at 1.8V with >80% of faradaic efficiency (FE), yielding 16.9 g H_2_/kg PET [93]. Another CuO nanowire electrocatalyst was also used to oxidize EG-to-formate with up to ∼86.5% selectivity and ∼88% of FE [94]. Afterward, Wang et al. [95] extended the electrocatalytic integrating strategy by combining electrocatalytic PET hydrolysate oxidation at anode (EG-to-FA) and electrocatalytic CO_2_ reduction at cathode (CO_2_/H_2_O-to-FA), with both electrodes generating FA. Moreover, NiCo_2_O_4_, used as an anode electrocatalyst, significantly enhanced selectivity for EG oxidation with a high FE of 90% and a current density of 20 mA/cm^2^ at 1.90 V. In the pathways of EG-to-formate/FA, oxidation of EG on anodic electrocatalysts is highly preferred for the generation of glyoxal intermediates and formate/FA in alkaline environments through C–C cleavage (Fig. 5c). Based on density functional theory calculation, EG oxidation on electrocatalyst surface tends to form ∗OCH_2_–CH_2_O∗ intermediate (an exothermic process) via O–H bond cleavage, followed with the conversion of ∗OCH_2_–CH_2_O∗ to ∗OCH–HCO∗ (an exothermic process) and ∗OCH–HCO∗ to FA via C–C bond cleavage (Fig. 5b) [94,95]. Another frequently reported electrocatalytic conversion pathway is EG-to-GlA in an alkaline atmosphere. An electrocatalyst of PdAg grown on nickel foam (PdAg/NF) has been developed to selectively oxidize EG-to-GlA, achieving 100% of FE and 300 mA/cm^2^ of current density at 1.02 V [96]. An Au/Ni(OH)_2_ was adopted as an anodic electrocatalyst to oxidize EG-to-GlA coupled with H_2_ production (11.2 g H_2_/kg PET), achieving 326.2 mA/cm^2^ of current densities at 1.15 V and 96% of FE [97]. In the pathway of EG-to-GlA, the reactions sequentially include electrooxidization of EG to glycolaldehyde by nucleophilic dehydrogenation, reversible conversion to enol in alkaline aqueous, and finally generation of GlA by nucleophilic dehydrogenation (Fig. 5c). Additionally, Shi et al. [98] illustrated the further electrocatalytic oxidation pathway of EG-to-carbonate using Pd/Ni foam as electrocatalyst, which exhibited excellent electrocatalytic activity (400 mA/cm^2^ at 0.7 V), high selectivity (95%), and FE (93%) for production of carbonate (CO_3_^2−^). Mechanism of C–C bond cleavage is proposed to promote alkaline EG oxidation, with glyoxal as an intermediate (Fig. 5c). Electroreforming of PET offers a sustainable and scalable route towards simultaneous plastic waste elimination, chemical synthesis, and fuel generation. Furthermore, developing efficient EG oxidation reactions with optimized electrocatalysts will broaden the upcycling prospect of PET wastes.

**Fig. 6:**
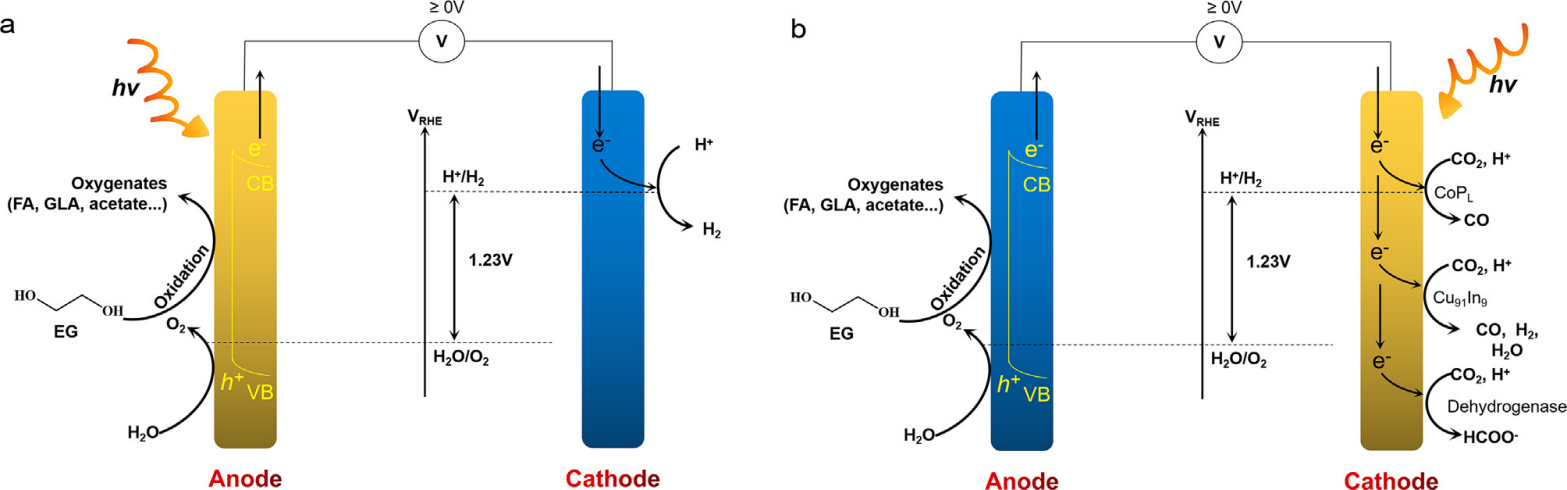
(a) Scheme of PEC reactions with anodic photoreforming and cathodic reduction [106]. (b) Scheme of PEC reactions with anodic reforming of PET and photocathdic CO_2_-to-fuel [103].

Photoelectrochemical (PEC) technology integrates the processes of photocatalysis and electrocatalysis to stimulate redox reactions in much milder environments. Mechanistically, a photoelectrode absorbs solar energy and transfers its photoexcited charge carriers to the catalytic center of redox sites, leading to solar-driven electricity or reduced voltage input by solar energy of PEC systems (Fig. 6a and b) [99]. Using photocatalysis with an external voltage, a PEC system was shown to photoreforming EG-to-FA via photoanode [Fe_2_O_3_/Ni(OH)_x_] and generated H_2_ on cathode [Ni(OH)_x_] at 3.5 mA/cm^2^, 1.2V [100]. Similarly, another PEC system used nano-Ni-P-TiO_2_ nanorods as the photoanode and carbon nanotubes/nanoNi-P as the cathode for the photoreforming of EG-to-FA and for generating H_2_, resulting in 100 mA/cm^2^ at 0.18 V with 100% of FE [101]. Moreover, many studies have developed solar-driven PEC systems that operate without an external voltage. A PEC system by Bhattacharjee et al. [102] was reported to convert EG-to-GLA on photoanodic Cu_30_Pd_70_ catalyst and generate H_2_ (0.75−1.28 mmol/cm^2^) on Pt cathode, with photocurrent density of 3.7 ± 0.3 to 5.1 ± 2.3 mA/cm^2^ without bias. Afterward, Bhattacharjee et al. [103] developed another PEC system (>90% of FE, 2.4 ± 0.3 mA/cm^2^ without bias) that reformed EG-to-GLA on anodic Cu_27_Pd_73_ catalyst, while perovskite-based photocathode enabled the integration of different CO_2_-reduction catalysts such as cobalt porphyrin, Cu_91_In_9_ alloy, and formate dehydrogenase enzyme to produce CO, syngas (CO and H_2_), and formate, respectively (Fig. 6b). PEC platforms enable the effective reforming of PET waste under mild conditions with renewable solar sources and with lower/no voltage. The selection and modification of catalysts provide reactive sites for redox activities, improving the selectivity of targeted products by manipulating the oxidizing ability of the photogenerated holes. Therefore, the PEC strategy provides a proof-of-concept alternative for photoreforming and electroreforming, approaching the performance and versatility required for the commercially viable waste utilization.

## 4 Tandem abiotic/biotic upcycling pathways of PET

Chemical approaches, in comparison, focus on selective depolymerization of PET streams in faster and more tolerant pathways, while biological processes use robust engineered bacterial strains to convert organic monomers to target products. Abiotic depolymerization breaks down PET wastes into TPA, BHET, and EG, which can be used as a substrate in enzymatic depolymerization. On the one hand, several chemo-enzymatic depolymerization processes have been developed, wherein chemical depolymerization yields oligomers, BHET, TPA, and EG, that can serve as substrates for bioconversion. Generally, hydrolysis is the most commonly adopted abiotic pathway of PET depolymerization, followed by engineered biological metabolism that promotes PET depolymerization as well as further bioconversion of primary products (e.g., TPA/EG) in open-loop pathways [32,40,42,99]. BHET, the main product from the glycolysis of PET, can also serve as a substrate for bioconversions. Gabriella et al. [105] developed an integrated glycolysis-enzymatic strategy to obtain TPA from PET within a short process timeframe (24 h), where PET was initially glycolyzed in the presence of a eutectic solvent-based catalyst and EG to obtain BHET (70% yield); subsequently, the recovered BHET was hydrolyzed by *Candida antarctica* lipase B to obtain TPA. Furthermore, Kim et al. [43] developed another integrated glycolysis-enzymatic/whole cells strategy, where PET was effectively depolymerized to TPA and EG through glycolysis of PET with betaine and the enzymatic hydrolysis for PET glycolysis slurry, then the recovered TPA and EG were converted by the whole cells of *E*.*coli* and *Gluconobacter oxydans* into PCA and GLA, respectively. Additionally, integrated biocatalytic PEC systems show great potential for sustainable redox biotransformation with electrons obtained from PET. A solar-driven PEC-biosynthetic system was demonstrated to convert EG-to-formate/acetate, owing to enhanced charge-separation dynamics, decreased charge-transfer resistance, suppressed charge recombination, and more upward band bending via Zr:α-Fe_2_O_3_ photoanode [106]. Meanwhile, its cathodes could activate redox enzymes of peroxygenase (oxyfunctionalization of C–H bonds), l-glutamate dehydrogenase (amination of C=O bonds), or ene-reductase (asymmetric hydrogenation of C=C bonds) (Fig. S3). In comparison, traditional PEC cells use semiconducting electrodes to directly convert sunlight into electric power or chemical fuels, while the PEC-biosynthetic systems integrate new concepts for solar-energy conversion using solid-state catalytic electrodes as solar-powered activators of enzymes. In contrast, pyrolysis is preferred for plastic mixture treatment due to the method's universality, capable of converting multiple polymeric wastes into usable products such as liquid fuels (i.e., aviation fuel, diesel, gasoline, aromatics), gaseous products (i.e., syngas, methane, H_2_), plastic monomers, and carbon nanotubes. Oxidative depolymerization of mixed PE, PS, and PET was fulfilled at 160−210 °C for 2−5 h; subsequently, the engineered *P. putida* funneled the heterogeneous mixture of oxygenates into β-ketoadipate or polyhydroxyalkanoates [104]. Further parameter adjustment and appropriate catalyst selection would allow pyrolysis to provide versatility in terms of product preferences and depolymerization efficiencies [83].

Considering the above cases, abiotic depolymerization of PET into TPA and EG (closed-loop recycling) (Section 3.1) can potentially cooperate with biotic upcycling pathways (Section 2.2), including EG-based, TPA-based, and TPA-EG-based bio-pathways for upcycling [41,50,107]. The tandem abiotic-biotic depolymerization process utilizes the advantages of abiotic approaches (flexibility, simplicity, fast, and controllable) and biotic approaches (selectivity and mild conditions) to produce TPA and EG, as well as high value-added chemicals.

## 5 Discussion and perspectives

**Fig. 7:**
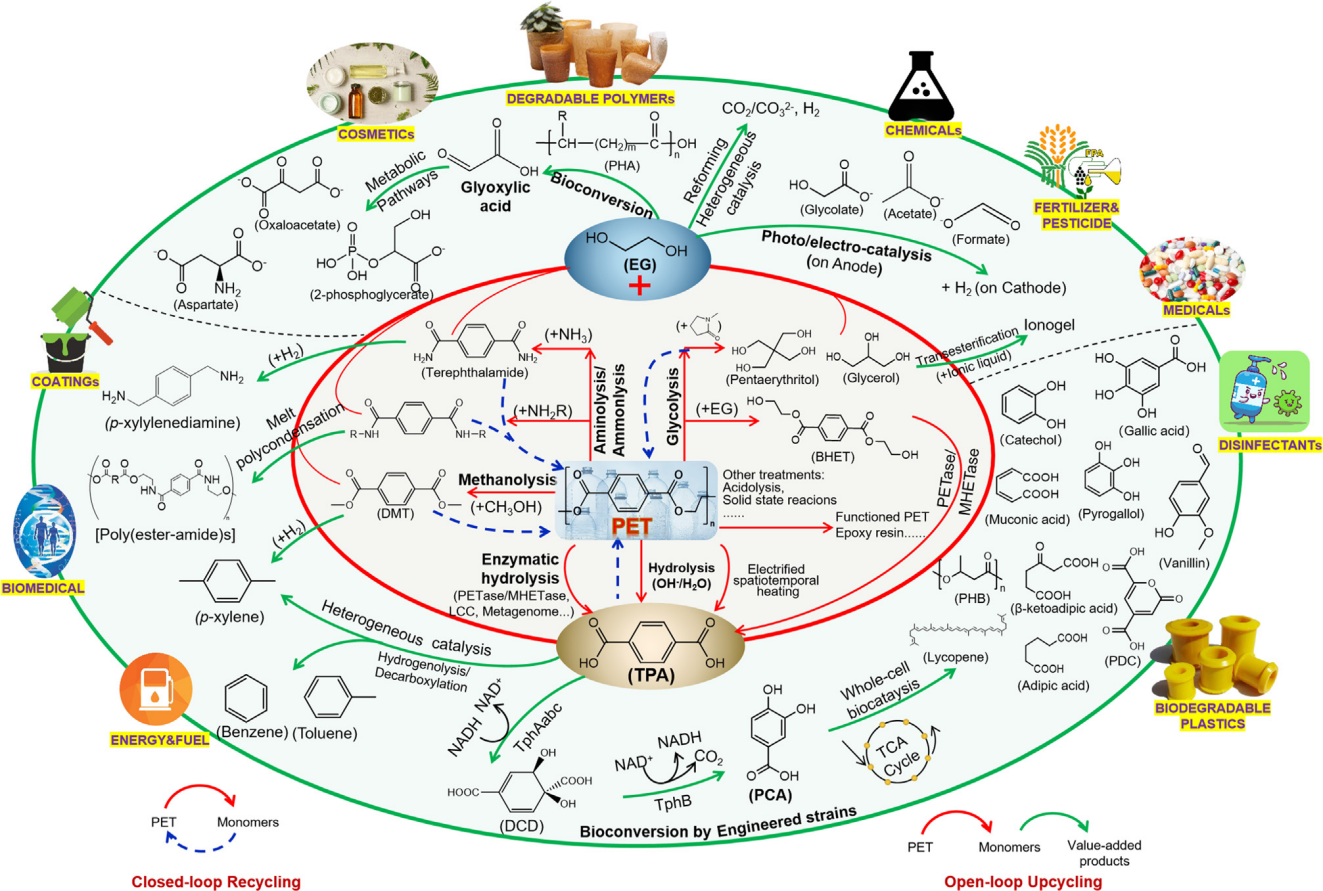
Overview of upcycling/recycling of PET via both biotic and abiotic pathways.

Plastic waste represents not only a global pollution problem, but also a carbon-rich, low-cost, globally available feedstock. This review comprehensively addressed the biotic and abiotic strategies for closed-loop recycling and open-loop upcycling of PET, focusing on the conversion pathways, intermediates/end-products, application fields, etc (Fig. 7). Remarkably, over 25 upgraded products have been derived from PET upcycling, with substantial applications (i.e., cosmetics, degradable polymers, medicals, disinfectants, fuels, and coatings) (Fig. 7). Abiotic recycling/upcycling of PET offers an imperative complement to traditional mechanical and solvent-based recycling approaches, towards a more holistic management strategy that can comprehensively depolymerize or convert PET in a more flexible and simple way [8,108]. Biotic recycling/upcycling of PET exhibits great promise for the development of eco-friendly approaches, with microbial recycling of PET potentially reducing CO_2_ emissions from PET waste treatments and achieving a 30%–40% reduction in CO_2_ emissions from landfills and incineration [109]. More opportunities can be derived from combining abiotic and biotic catalysis to generate commodity chemicals and alternative materials, ideally at lower energy inputs, greenhouse gas emissions, and costs, compared to virgin polymer fabrication [110]. These developments reveal the great potential of PET upcycling in promoting a global circular economy and resource efficiency. More efforts can be made to increase the amenability to catalytic deconstruction in abiotic strategies, improve the microbial depolymerization of PET (e.g., hydrolysis efficiency, enzymatic activity, thermal and pH level stability), and develop new microorganisms or hydrolases capable of degrading PET through computational and machine learning algorithms [15,24].

From the perspective of biotic pathways, PET depolymerization is primarily illustrated with PETases (i.e., cutinases and *Is*PETase), specifically producing TPA and EG. Further biological/chemical conversion on base monomers is required for upcycling purposes. For PET upcycling, TPA can be converted into various aromatics or acids via TCA cycle or chemical catalysis, and EG can be converted into glyoxylic acid via whole-cell bioconversion or into formate, acetate, or glycolate via chemical oxidation. To achieve the bio-upcycling of plastics, efforts should focus on discovering and optimizing enzymatic depolymerases, metabolic pathways, and microbial catalysts for converting plastic monomers. Apart from PETases as the primary enzyme for PET depolymerization, the subsequent hydrolases of BHETase and MHETase have recently received increasing attention. As reported, newly engineered BHETases (ChryBHETase and BsEst) enhanced the catalytic efficiency by up to 3.5-fold compared to the wild-type [17]. The coupled BHETase with PETases (i.e., FAST-PETase, DuraPETase, ThermoPETase, LCC) into a two-enzyme system significantly achieved up to 7.0-fold improved TPA production than the state-of-the-art PET hydrolases [17]. The realization of plastic bio-upcycling would open up a broad spectrum of value-added products, offering better end-of-life solutions for various plastics and mixtures.

From the perspective of abiotic pathways, thermal treatment is one of the most common abiotic methods to recycle/upcycle plastics into carbon-based materials or valuable products. During thermal treatment, PET breaks down mainly through C–O and C–C homolytic cleavage [111]. Thermal treatments of PET mainly include hydrolysis (→TPA + EG), glycolysis (→BHET), aminolysis/ammonlysis (→TPA-NH + EG), and methanolysis (→DMT + EG), mostly conducted under 180−300 °C and 0.1−3.0 MPa. Photocatalysis and/or electrocatalysis have been explored as renewable technologies using energy from protons or electrons, respectively, to accelerate plastic upcycling, allowing milder reaction conditions and enabling selective chemistry on plastic conversion compared to thermal techniques. Photo/electrocatalysis of PET leads to oxidative depolymerization processes. The monomer EG can serve as the direct electronic donor, being oxidized to GA, FA, or acetate, while simultaneously generating H_2_ that can serve as a reductant. Meanwhile, combined photocatalysis and electrocatalysis synergistically promote the oxidation of PET to value-added products. However, TPA is seldom chemically redoxed, while microbial conversion via the β-ketoadipate pathway favorably converts it. Future work should consider comprehensive factors in practical scenarios, including catalyst efficacy (conversion, selectivity, and stability), substrate solubility, reactor design, and cost. Furthermore, green and sustainable PEC systems show great potential in the economical upcycling of PET since the simultaneous employment of light and electrical energy tunes the directionality and the kinetics of photoexcited electrons for efficient activation of redox-active sites. PEC platforms should pursue more swiftness, scalability, and sustainability for improvement.

Moreover, since the significant accumulation of plastics has already existed, developing user-friendly, waste-free, energy-efficient, and sustainable upcycling technologies for treating plastic waste on an industrial scale is essential for protecting global ecosystems. For PET upcycling, efforts can be dedicated to developing specific PETases with thermostable, fast-degradable, and durable abilities or to developing advanced chemical technologies carried out in milder conditions, probably with sustainability from solar energy or electricity. The development of cost-effective and environmentally friendly upcycling technologies for mixed plastics should also be focused on because of the global difficulties in plastic sorting. More commonalities and differences in molecule characters (i.e., composites, structure, and bonds) among different plastics should be discovered and verified to explore the possible upcycling approaches through similar, cooperated, complementary, or progressive principles or theories. In view of eco-environmental and economic effects, PET has a high potential to reduce energy demands, probably with significant breakthroughs in recycling/upcycling technologies towards carbon neutrality solutions. When recycled/upcycled PET achieves competitive pricing and becomes carbon-negative, it emerges as a viable alternative material for packaging or chemical reagents. Increasing the scale of PET usage not only improves its proportion in the plastic recycling stream, but also improves the ability for plastic sorting and reusing [112]. PET recycling/upcycling is beneficial for the eco-environment even with fossil fuel-based polymers due to the aforementioned lower carbon footprint.

## CRediT authorship contribution statement

J.Q.Y.: investigation, data arrange, writing–original draft, funding acquisition. Z.L.L.: conceptualization, writing, funding acquisition. Q.Y.X.: data curation, figures. W.Z.L.: data curation, figures, revision. S.H.G.: revision, data curation. P.W.Q.: data curation, supervision. Z.L.C.: investigation, data arrange, writing–original draft. A.J.W.: conceptualization, supervision.

## Declaration of competing interests

The authors declare that they have no known competing financial interests or personal relationships that could have appeared to influence the work reported in this paper.
