# Supplementary material for: Towards carbon neutrality: Sustainable recycling and upcycling strategies and mechanisms for polyethylene terephthalate via biotic/abiotic pathways
Source: Eco Environ Health. 2024 Feb 27;3(2):117–30. doi: 10.1016/j.eehl.2024.01.010 (PMC11021832; doi:10.1016/j.eehl.2024.01.010)
Supplement: Multimedia component 1 [file mmc1.docx]

**Supplementary Information:**

**Towards Carbon Neutrality: Sustainable Recycling and Upcycling Strategies and mechanisms for Polyethylene Terephthalate via Biotic/Abiotic Pathways**

Jiaqi Yang^a^, Zhiling Li^b*^, Qiongying Xu^a^, Wenzong Liu^a^, Shuhong Gao^a^, Peiwu Qin^c,d^, Zhenglin Chen^c,d**^, Aijie Wang^a,b^

^a^ School of Civil & Environmental Engineering, Harbin Institute of Technology (Shenzhen), Shenzhen 518055, China;

^b^ State Key Laboratory of Urban Water Resources and Environment, School of Environment, Harbin Institute of Technology, Harbin 150090, China;

^c^ Institute of Biopharmaceutical and Health Engineering, Shenzhen International Graduate School, Tsinghua University, Shenzhen, Guangdong 518055, China

^d^ Tsinghua-Berkeley Shenzhen Institute, Tsinghua Shenzhen International Graduate School, Tsinghua University, Shenzhen 518055, China

Table S1 Detailed PET upcycling strategies mainly in opened-loop pathways

| No. | Biotic/  Abiotic | Main Approaches | Operations/Processes | PET conversion Products/  Applications | Ref. |
| --- | --- | --- | --- | --- | --- |
| 1 | Biotic | Microbiome bioconversion |   (Ethylene Glycol Metabolism by *Pseudomonas putida*) | Glyoxylic acid | [1,2] |
| 2 | Biotic | Microbiome bioconversion |  | Glyoxylate,  2-phosphoglycerate, Aspartate, Oxaloacetate | [3] |
| 3 | Biotic | Microbiome bioconversion  (As carbon sources) |  | Biodegradable plastic polyhydroxyalkanoate (PHA) | [4] |
| 4 | Biotic | Enzymatic/  microbiome bioconversion |  | Vanillin | [5] |
| 5 | Biotic | Enzymatic  (*Is*PETase)  /Whole-cell biocatalyst, Hydrophobins adsorption |  | MHET, BHET, TPA | [6] |
| 6 | Abiotic | Electrified spatiotemporal heating |  | TPA | [7] |
| 7 | Abiotic | Alkaline Hydrolysis |  | H_2_(fuel),  Na_2_-TPA,  Na_2_CO_3_ | [8] |
| 8 | Abiotic | Glycolysis (180℃)  Transesterification reactions |  | Ionogels  (Containing depolymerized PET oligomers, N-methyl-2-pyrrolidone, ionic liquids) | [9] |
| 9 | Abiotic | Methanolysis |  | EG,  p-xylene  (fuel) | [10] |
| 10 | Abiotic | Acidolysis |  | TPA, epoxy resin | [11] |
| 11 | Abiotic | Glycolysis |   Solid-state reaction at 240℃ | Antibacterial PET | [12] |
| 12 | Abiotic | Aminolysis,  Melt polycondensation |  | Poly(ester-amide)s  (Material for Biomedical sectors) | [13] |
| 13 | Abiotic | Alkaline Hydrolysis,  Photoreforming |  | TPA, H_2_, formate, acetate... | [14,15] |
| 14 | Abiotic | Alkaline Hydrolysis,  Photoreforming |  | TPA, H_2_, formate, acetate... | [16,17] |
| 15 | Abiotic | Alkaline Hydrolysis,  Photoreforming | 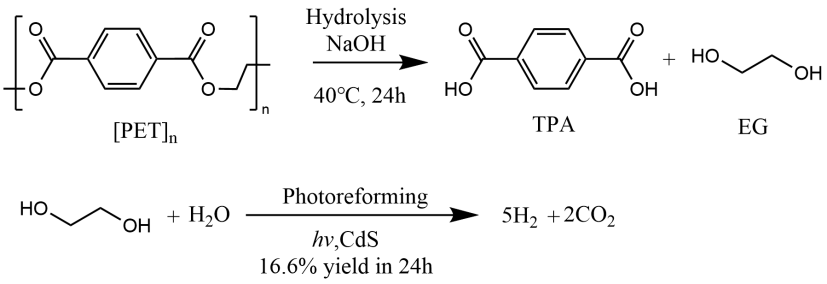 | TPA, EG, CO_2_, H_2_ | [14] |
| 16 | Abiotic | Alkaline Hydrolysis,  Photoforming |   CPDs-CN: carbonized polymer dotsgraphitic carbon nitride | TPA, glycolic acid, glycolaldehyde, ethanol, fomic acid, acetic acid, H_2_ | [18] |
| 17 | Abiotic | Alkaline Hydrolysis, electrolysis |   current density of 500 mA·cm^-2^ at 1.8V, >80% of FE, 16.9gH_2_/kg_PET_ | H_2_ (Fuel),  TPA,  potassium diformate | [19] |
| 18 | Abiotic | Alkaline Hydrolysis,  Electrocatalysis |   current density of 20 mA·cm^-2^ at 1.90V, >90% of FE | HCOOH, TPA | [20] |
| 19 | Abiotic | Alkaline Hydrolysis,  Electrocatalysis |   current densities of 326.2 mA·cm^-2^ at 1.15 V, 96% of FE, 11.2gH_2_/kg_PET_. | Glycolic acid, TPA, H_2_ | [21] |
| 20 | Abiotic | Alkaline Hydrolysis,  Photoelectro-  catalysis |    | TPA, Glycolic acid, H_2_ | [22] |
| 21 | Abiotic | Alkaline Hydrolysis,  Photoelectro-  catalysis |    | TPA, Glycolic acid,  CO, syngas, or formate | [23] |
| 22 | Abiotic | Alkaline Hydrolysis,  Photoelectro-  catalysis |  | TPA, Formic acid, H_2_ | [24] |
| 23 | Abiotic | Alkaline Hydrolysis,  Photoelectro-  catalysis |  | TPA, Formic acid, H_2_ | [25] |
| 24 | Abiotic | Alkaline Hydrolysis, Photoelectro-  catalysis | 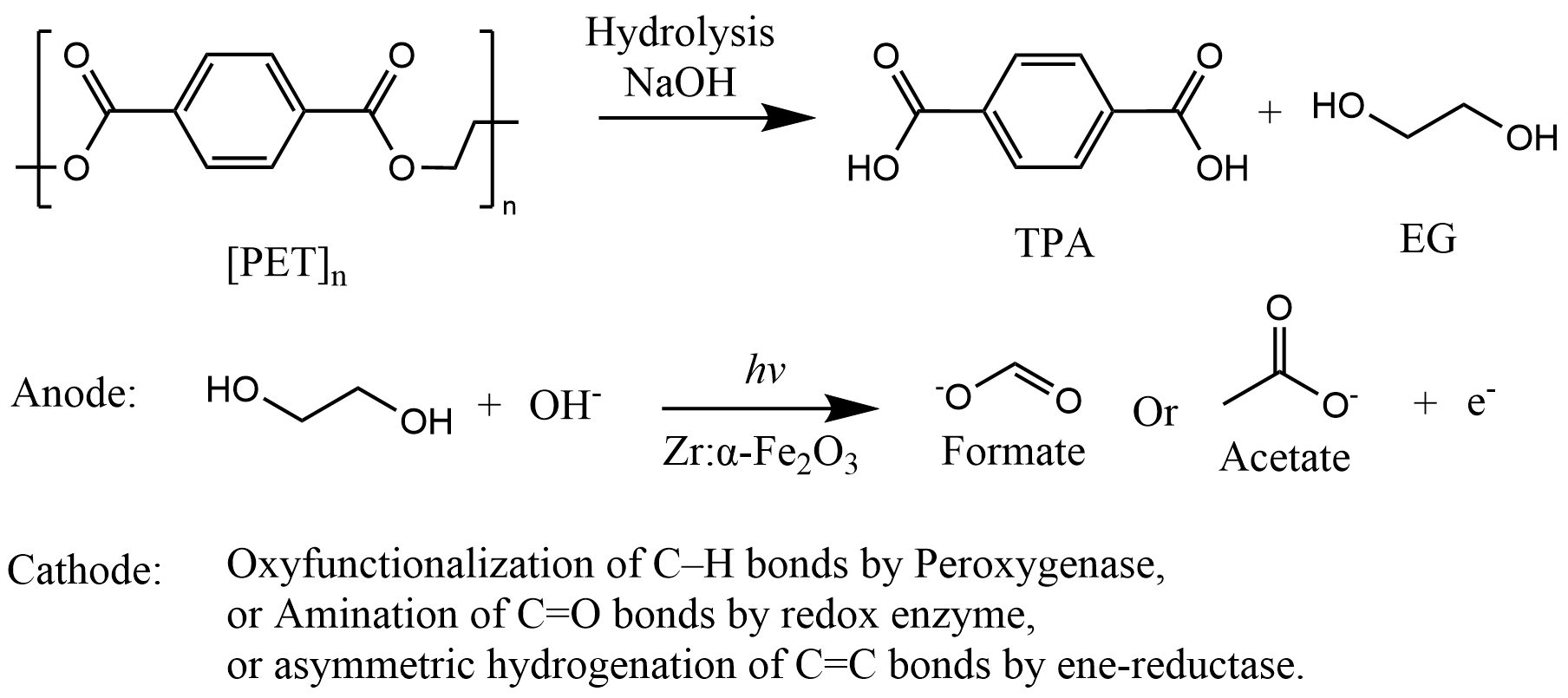 | HOOC-COOR，  R-COOH，  TPA，  Biosynthesis on cathode | [26] |
| 25 | Abiotic | Hydrolysis, reforming, hydrogenolysis/  decarboxylation |  | Benzene, Toluene, p-xylene | [27] |
| 26 | Abiotic  /Biotic | Hydrolysis, Engineered microbiome bioconversion |  | Protocatechuic acid | [28] |
| 27 | Abiotic  /Biotic | Glycolysis,  Enzymatic hydrolysis,  Cell biotransformation |    | Catechol (coating agents) | [29] |
| 28 | Abiotic  /Biotic | Microwave hydrolysis,  Microbiome conversion | 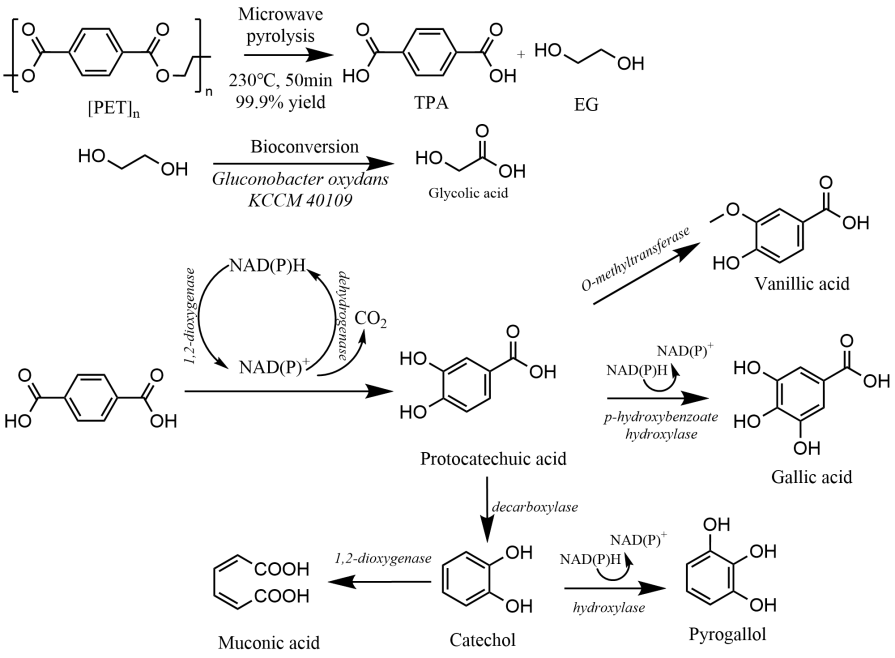 | Glycolic acid,  Gallic acid,  Vanillic acid,  Pyrogallol,  Catechol,  Muconic acid. | [30,31] |
| 29 | Abiotic  /Biotic | Alkaline Hydrolysis,  Microbial bioconversion | 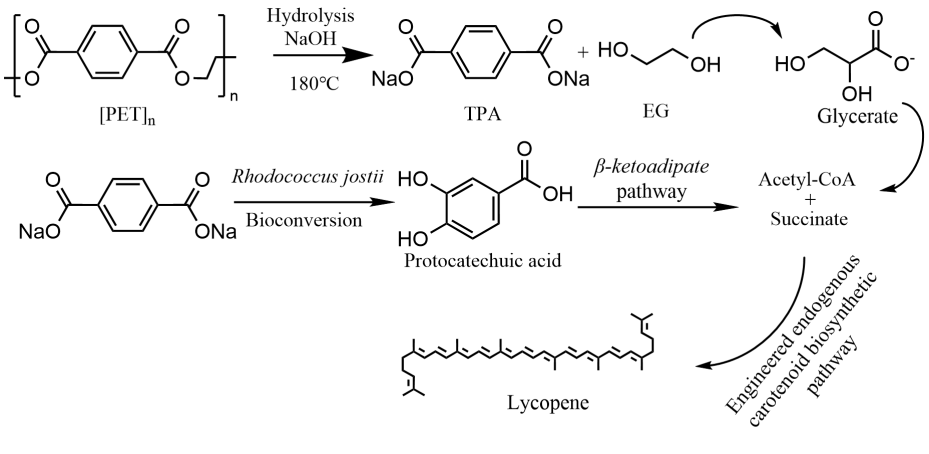 | Lycopene (medical) | [32] |
| 30 | Abiotic  /Biotic | Glycolysis,  Microbial bioconversion | 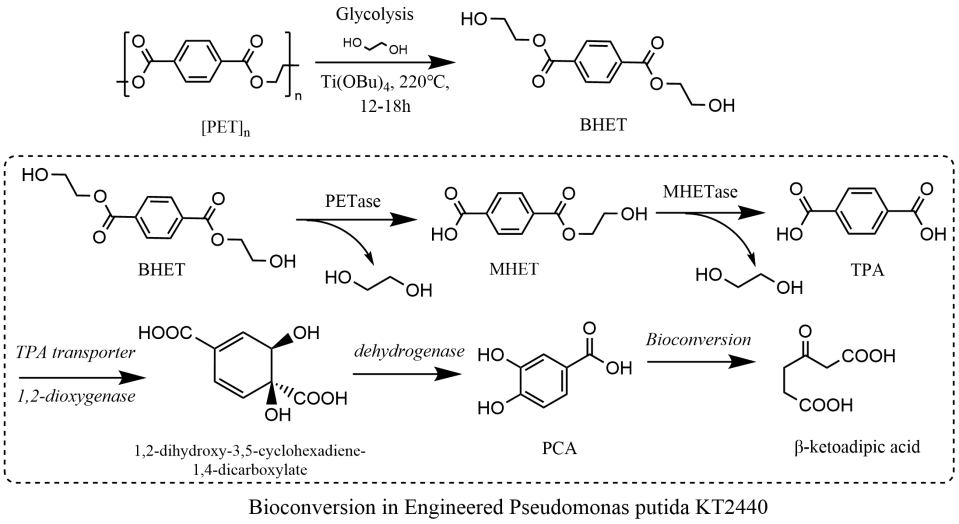 | β-ketoadipic acid (bioproduct that can be polymerized into a nylon-6,6 analog) | [33,34] |
| 31 | Abiotic  /Biotic | Microwave- assisted hydrolysis, Engineered whole-cell biocatalysts |  | 2-pyrone-4,6-dicarboxylic acid (PDC) | [35] |
| 32 | Abiotic  /Biotic | Co-cultivation system of Engineered whole-cell biocatalysts | 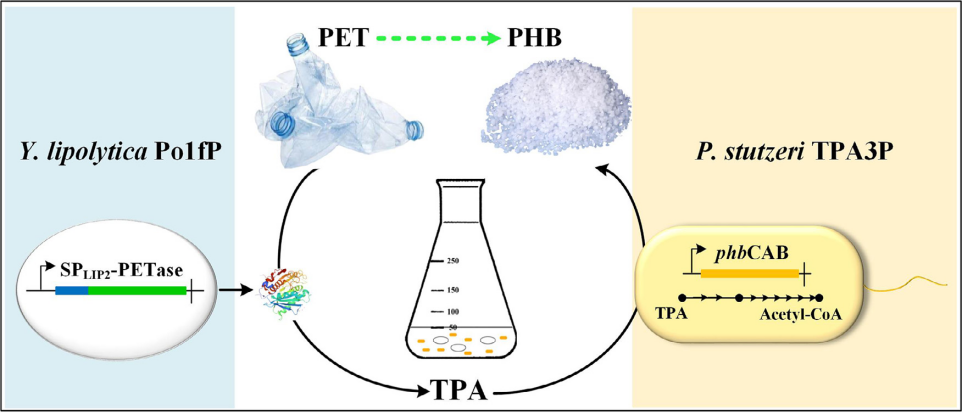 | PHB | [36] |

**Figures**


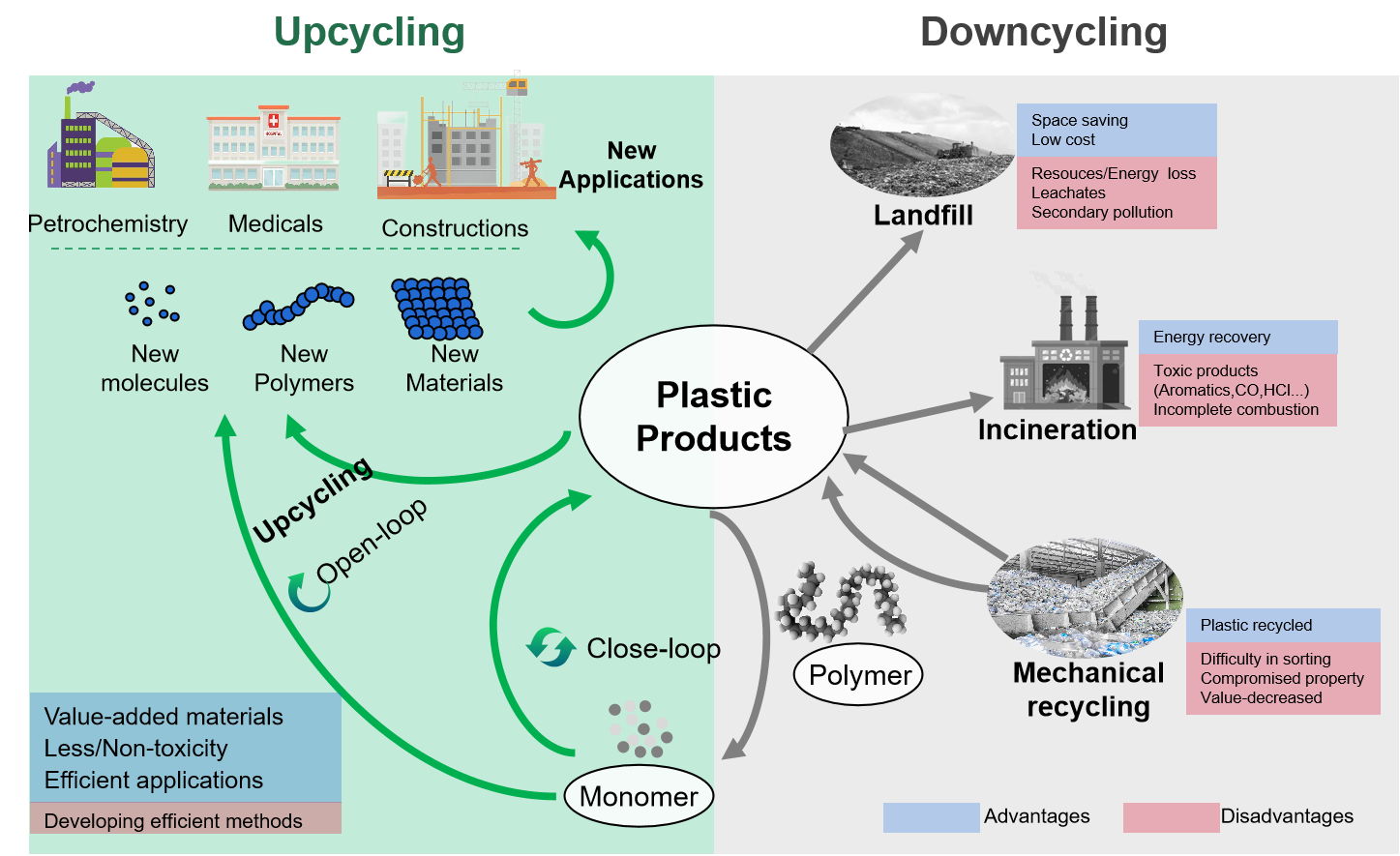


Fig. S1 Pathways of upcycling and downcycling of plastics

Fig. S2 Thermochemical depolymerization processes (hydrolysis, glycolysis, methanolysis, aminolysis, ammonolysis) of PET in closed-loop recycling


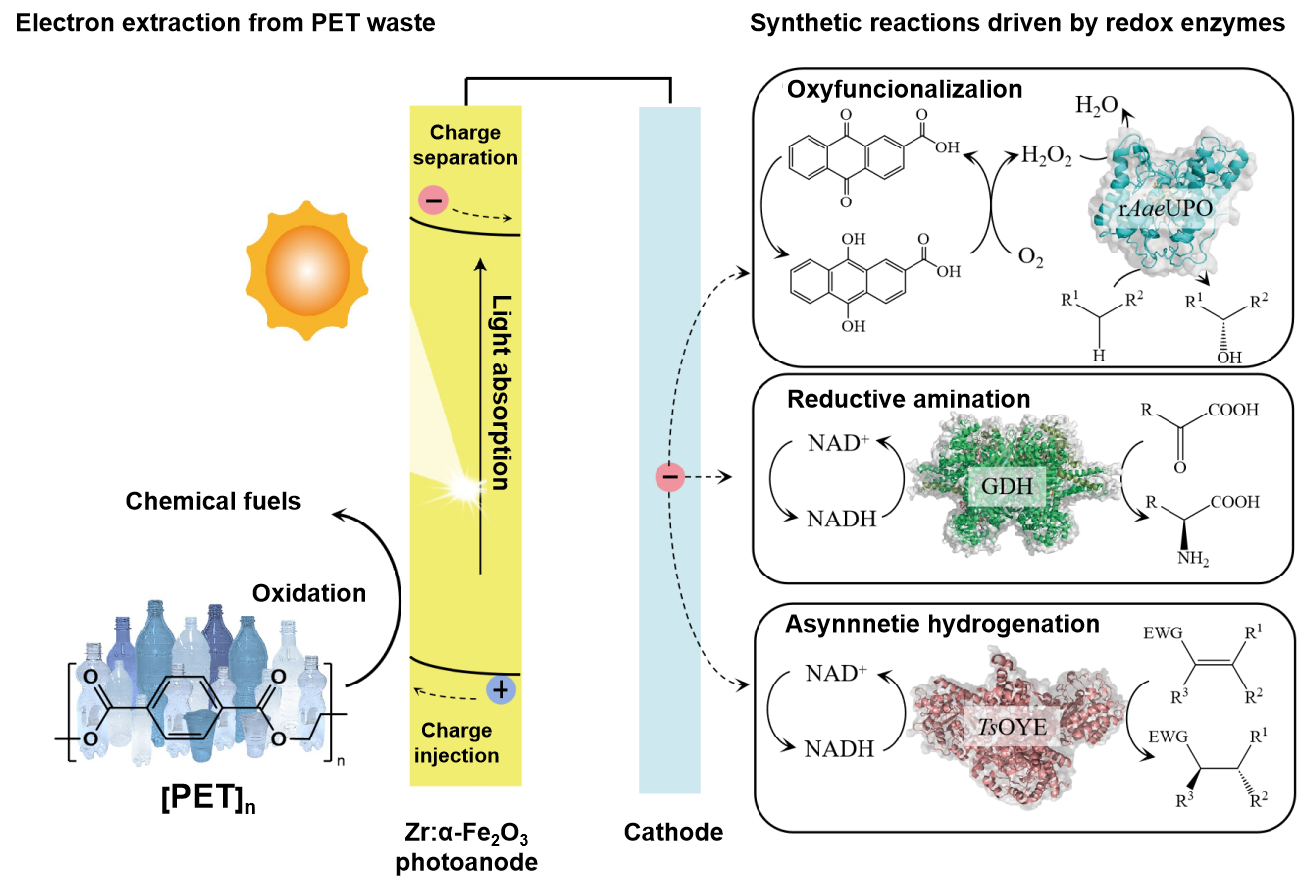


Fig. S3 Scheme of PEC-biosynthetic reactions using PET as electron donors. Cathodes reduce O_2_ to H_2_O_2_ for biocatalytic oxyfunctionalization and NAD^+^ to NADH for enzymatic amination and asymmetric hydrogenation. Reproduced with permission from ref [([26])](javascript:void(0);). Copyright 2022 Springer Nature.

**Reference**

[1] N.-K. Kim, S.-H. Lee, H.-D. Park, Current biotechnologies on depolymerization of polyethylene terephthalate (PET) and repolymerization of reclaimed monomers from PET for bio-upcycling: A critical review, Bioresource Technology 363 (2022) 127931. https://doi.org/10.1016/j.biortech.2022.127931.

[2] B. Mückschel, O. Simon, J. Klebensberger, N. Graf, B. Rosche, J. Altenbuchner, J. Pfannstiel, A. Huber, B. Hauer, Ethylene Glycol Metabolism by Pseudomonas putida, Applied and Environmental Microbiology 78 (2012) 8531–8539. https://doi.org/10.1128/AEM.02062-12.

[3] S. von Borzyskowski, H. Schulz-Mirbach, M. Troncoso Castellanos, F. Severi, P.A. Gómez-Coronado, N. Paczia, T. Glatter, A. Bar-Even, S.N. Lindner, T.J. Erb, Implementation of the β-hydroxyaspartate cycle increases growth performance of Pseudomonas putida on the PET monomer ethylene glycol, Metabolic Engineering 76 (2023) 97–109. https://doi.org/10.1016/j.ymben.2023.01.011.

[4] T. Narancic, M. Salvador, G.M. Hughes, N. Beagan, U. Abdulmutalib, S.T. Kenny, H. Wu, M. Saccomanno, J. Um, K.E. O’Connor, J.I. Jiménez, Genome analysis of the metabolically versatile Pseudomonas umsongensis GO16: the genetic basis for PET monomer upcycling into polyhydroxyalkanoates, Microbial Biotechnology 14 (2021) 2463–2480. https://doi.org/10.1111/1751-7915.13712.

[5] J.C. Sadler, S. Wallace, Microbial synthesis of vanillin from waste poly(ethylene terephthalate), Green Chem. 23 (2021) 4665–4672. https://doi.org/10.1039/D1GC00931A.

[6] Z. Chen, R. Duan, Y. Xiao, Y. Wei, H. Zhang, X. Sun, S. Wang, Y. Cheng, X. Wang, S. Tong, Y. Yao, C. Zhu, H. Yang, Y. Wang, Z. Wang, Biodegradation of highly crystallized poly(ethylene terephthalate) through cell surface codisplay of bacterial PETase and hydrophobin, Nat Commun 13 (2022) 7138. https://doi.org/10.1038/s41467-022-34908-z.

[7] Q. Dong, A.D. Lele, X. Zhao, S. Li, S. Cheng, Y. Wang, M. Cui, M. Guo, A.H. Brozena, Y. Lin, T. Li, L. Xu, A. Qi, I.G. Kevrekidis, J. Mei, X. Pan, D. Liu, Y. Ju, L. Hu, Depolymerization of plastics by means of electrified spatiotemporal heating, Nature 616 (2023) 488–494. https://doi.org/10.1038/s41586-023-05845-8.

[8] H. Su, T. Li, S. Wang, L. Zhu, Y. Hu, Low-temperature upcycling of PET waste into high-purity H2 fuel in a one-pot hydrothermal system with in situ CO2 capture, Journal of Hazardous Materials 443 (2023) 130120. https://doi.org/10.1016/j.jhazmat.2022.130120.

[9] Z. Guo, J.S.K. Lim, K.W.J. Ng, W. Yan, N. Gupta, X.M. Hu, Direct One-Step Controlled Partial Depolymerization and Upcycling of Poly(ethylene terephthalate) into Hyperbranched Oligomeric Ionogel, ACS Sustainable Chem. Eng. 11 (2023) 1394–1404. https://doi.org/10.1021/acssuschemeng.2c05911.

[10] Z. Gao, B. Ma, S. Chen, J. Tian, C. Zhao, Converting waste PET plastics into automobile fuels and antifreeze components, Nat Commun 13 (2022) 3343. https://doi.org/10.1038/s41467-022-31078-w.

[11] C.N. Hoang, N.T. Nguyen, S.T. Ta, N.N. Nguyen, D. Hoang, Acidolysis of Poly(ethylene terephthalate) Waste Using Succinic Acid under Microwave Irradiation as a New Chemical Upcycling Method, ACS Omega 7 (2022) 47285–47295. https://doi.org/10.1021/acsomega.2c06642.

[12] H. Zhang, T. Fang, X. Yao, X. Li, W. Zhu, Catalytic Amounts of an Antibacterial Monomer Enable the Upcycling of Poly(Ethylene Terephthalate) Waste, Advanced Materials n/a (n.d.) 2210758. https://doi.org/10.1002/adma.202210758.

[13] J.M. Payne, M. Kamran, M.G. Davidson, M.D. Jones, Versatile Chemical Recycling Strategies: Value-Added Chemicals from Polyester and Polycarbonate Waste, ChemSusChem 15 (2022) e202200255. https://doi.org/10.1002/cssc.202200255.

[14] U. Taylor, H. Kasap, E. Reisner, Photoreforming of Nonrecyclable Plastic Waste over a Carbon Nitride/Nickel Phosphide Catalyst, J. Am. Chem. Soc. 141 (2019) 15201–15210. https://doi.org/10.1021/jacs.9b06872.

[15] T. Uekert, M.F. Kuehnel, D.W. Wakerley, E. Reisner, Plastic waste as a feedstock for solar-driven H2 generation, Energy Environ. Sci. 11 (2018) 2853–2857. https://doi.org/10.1039/C8EE01408F.

[16] B. Cao, S. Wan, Y. Wang, H. Guo, M. Ou, Q. Zhong, Highly-efficient visible-light-driven photocatalytic H2 evolution integrated with microplastic degradation over MXene/ZnxCd1-xS photocatalyst, Journal of Colloid and Interface Science 605 (2022) 311–319. https://doi.org/10.1016/j.jcis.2021.07.113.

[17] Y. Li, S. Wan, C. Lin, Y. Gao, Y. Lu, L. Wang, K. Zhang, Engineering of 2D/2D MoS2/CdxZn1−xS Photocatalyst for Solar H2 Evolution Coupled with Degradation of Plastic in Alkaline Solution, Solar RRL 5 (2021) 2000427. https://doi.org/10.1002/solr.202000427.

[18] M. Han, S. Zhu, C. Xia, B. Yang, Photocatalytic upcycling of poly(ethylene terephthalate) plastic to high-value chemicals, Applied Catalysis B: Environmental 316 (2022) 121662. https://doi.org/10.1016/j.apcatb.2022.121662.

[19] H. Zhou, Y. Ren, Z. Li, M. Xu, Y. Wang, R. Ge, X. Kong, L. Zheng, H. Duan, Electrocatalytic upcycling of polyethylene terephthalate to commodity chemicals and H2 fuel, Nat Commun 12 (2021) 4679. https://doi.org/10.1038/s41467-021-25048-x.

[20] J. Wang, X. Li, M. Wang, T. Zhang, X. Chai, J. Lu, T. Wang, Y. Zhao, D. Ma, Electrocatalytic Valorization of Poly(ethylene terephthalate) Plastic and CO2 for Simultaneous Production of Formic Acid, ACS Catal. 12 (2022) 6722–6728. https://doi.org/10.1021/acscatal.2c01128.

[21] Y. Yan, H. Zhou, S.-M. Xu, J. Yang, P. Hao, X. Cai, Y. Ren, M. Xu, X. Kong, M. Shao, Z. Li, H. Duan, Electrocatalytic Upcycling of Biomass and Plastic Wastes to Biodegradable Polymer Monomers and Hydrogen Fuel at High Current Densities, J. Am. Chem. Soc. 145 (2023) 6144–6155. https://doi.org/10.1021/jacs.2c11861.

[22] S. Bhattacharjee, V. Andrei, C. Pornrungroj, M. Rahaman, C.M. Pichler, E. Reisner, Reforming of Soluble Biomass and Plastic Derived Waste Using a Bias-Free Cu30Pd70|Perovskite|Pt Photoelectrochemical Device, Advanced Functional Materials 32 (2022) 2109313. https://doi.org/10.1002/adfm.202109313.

[23] S. Bhattacharjee, M. Rahaman, V. Andrei, M. Miller, S. Rodríguez-Jiménez, E. Lam, C. Pornrungroj, E. Reisner, Photoelectrochemical CO2-to-fuel conversion with simultaneous plastic reforming, Nat. Synth 2 (2023) 182–192. https://doi.org/10.1038/s44160-022-00196-0.

[24] C.-Y. Lin, S.-C. Huang, Y.-G. Lin, L.-C. Hsu, C.-T. Yi, Electrosynthesized Ni-P nanospheres with high activity and selectivity towards photoelectrochemical plastics reforming, Applied Catalysis B: Environmental 296 (2021) 120351. https://doi.org/10.1016/j.apcatb.2021.120351.

[25] X. Li, J. Wang, T. Zhang, T. Wang, Y. Zhao, Photoelectrochemical Catalysis of Waste Polyethylene Terephthalate Plastic to Coproduce Formic Acid and Hydrogen, ACS Sustainable Chem. Eng. 10 (2022) 9546–9552. https://doi.org/10.1021/acssuschemeng.2c02244.

[26] J. Kim, J. Jang, T. Hilberath, F. Hollmann, C.B. Park, Photoelectrocatalytic biosynthesis fuelled by microplastics, Nat. Synth 1 (2022) 776–786. https://doi.org/10.1038/s44160-022-00153-x.

[27] S. Lu, Y. Jing, B. Feng, Y. Guo, X. Liu, Y. Wang, H2-free Plastic Conversion: Converting PET back to BTX by Unlocking Hidden Hydrogen, ChemSusChem 14 (2021) 4242–4250. https://doi.org/10.1002/cssc.202100196.

[28] W.M. Kincannon, M. Zahn, R. Clare, J. Lusty Beech, A. Romberg, J. Larson, B. Bothner, G.T. Beckham, J.E. McGeehan, J.L. DuBois, Biochemical and structural characterization of an aromatic ring–hydroxylating dioxygenase for terephthalic acid catabolism, Proceedings of the National Academy of Sciences 119 (2022) e2121426119. https://doi.org/10.1073/pnas.2121426119.

[29] H.T. Kim, M. Hee Ryu, Y.J. Jung, S. Lim, H.M. Song, J. Park, S.Y. Hwang, H.-S. Lee, Y.J. Yeon, B.H. Sung, U.T. Bornscheuer, S.J. Park, J.C. Joo, D.X. Oh, Chemo-Biological Upcycling of Poly(ethylene terephthalate) to Multifunctional Coating Materials, ChemSusChem 14 (2021) 4251–4259. https://doi.org/10.1002/cssc.202100909.

[30] D.H. Kim, D.O. Han, K. In Shim, J.K. Kim, J.G. Pelton, M.H. Ryu, J.C. Joo, J.W. Han, H.T. Kim, K.H. Kim, One-Pot Chemo-bioprocess of PET Depolymerization and Recycling Enabled by a Biocompatible Catalyst, Betaine, ACS Catal. 11 (2021) 3996–4008. https://doi.org/10.1021/acscatal.0c04014.

[31] H.T. Kim, J.K. Kim, H.G. Cha, M.J. Kang, H.S. Lee, T.U. Khang, E.J. Yun, D.-H. Lee, B.K. Song, S.J. Park, J.C. Joo, K.H. Kim, Biological Valorization of Poly(ethylene terephthalate) Monomers for Upcycling Waste PET, ACS Sustainable Chem. Eng. 7 (2019) 19396–19406. https://doi.org/10.1021/acssuschemeng.9b03908.

[32] J. Diao, Y. Hu, Y. Tian, R. Carr, T.S. Moon, Upcycling of poly(ethylene terephthalate) to produce high-value bio-products, Cell Reports 42 (2023) 111908. https://doi.org/10.1016/j.celrep.2022.111908.

[33] A.Z. Werner, R. Clare, T.D. Mand, I. Pardo, K.J. Ramirez, S.J. Haugen, F. Bratti, G.N. Dexter, J.R. Elmore, J.D. Huenemann, G.L. Peabody, C.W. Johnson, N.A. Rorrer, D. Salvachúa, A.M. Guss, G.T. Beckham, Tandem chemical deconstruction and biological upcycling of poly(ethylene terephthalate) to β-ketoadipic acid by Pseudomonas putida KT2440, Metabolic Engineering 67 (2021) 250–261. https://doi.org/10.1016/j.ymben.2021.07.005.

[34] S. Yoshida, K. Hiraga, T. Takehana, I. Taniguchi, H. Yamaji, Y. Maeda, K. Toyohara, K. Miyamoto, Y. Kimura, K. Oda, A bacterium that degrades and assimilates poly(ethylene terephthalate), Science 351 (2016) 1196–1199. https://doi.org/10.1126/science.aad6359.

[35] M.J. Kang, H.T. Kim, M.-W. Lee, K.-A. Kim, T.U. Khang, H.M. Song, S.J. Park, J.C. Joo, H.G. Cha, A chemo-microbial hybrid process for the production of 2-pyrone-4,6-dicarboxylic acid as a promising bioplastic monomer from PET waste, Green Chem. 22 (2020) 3461–3469. https://doi.org/10.1039/D0GC00007H.

[36] P. Liu, T. Zhang, Y. Zheng, Q. Li, T. Su, Q. Qi, Potential one-step strategy for PET degradation and PHB biosynthesis through co-cultivation of two engineered microorganisms, Engineering Microbiology 1 (2021) 100003. https://doi.org/10.1016/j.engmic.2021.100003.
